# Aspergillus Mycotoxins: The Major Food Contaminants

**DOI:** 10.1002/advs.202412757

**Published:** 2025-02-07

**Authors:** Mengyao Xue, Zheng Qu, Antonio Moretti, Antonio F. Logrieco, Haiyan Chu, Qi Zhang, Changpo Sun, Xianfeng Ren, Li Cui, Qinglin Chen, Yi An, Chengjun Li, Huan Zhong, Zhiyan Cao, Feng Wang, Yuebing Sun, Lili Wang, Jie Hou, Chenchen Zhang, Mengmeng Yang, Yiming Ding, Yanpo Yao, Peiwu Li, Yong‐Guan Zhu

**Affiliations:** ^1^ Agro‐Environmental Protection Institute Ministry of Agriculture and Rural Affairs Tianjin 300191 China; ^2^ Xianghu Laboratory Zhejiang Provincial Laboratory of Agriculture Hangzhou 311231 China; ^3^ Institute of Sciences of Food Production National Research Council Bari 70126 Italy; ^4^ State Key Laboratory of Soil and Sustainable Agriculture Institute of Soil Science Chinese Academy of Science Nanjing China; ^5^ Oil Crops Research Institute Chinese Academy of Agricultural Sciences Wuhan 430062 China; ^6^ Academy National Food and Strategic Reserves Administration Beijing 100037 China; ^7^ Institute of Agricultural Quality Standards and Testing Technology Shandong Academy of Agricultural Sciences Jinan 250100 China; ^8^ Institute of Urban Environment Chinese Academy of Sciences Xiamen 361021 China; ^9^ Institute of Environmental Research at Greater Bay Area Guangzhou University Guangzhou 510006 China; ^10^ School of Environment Nanjing University Nanjing 210023 China; ^11^ College of Plant Protection Hebei Agricultural University Baoding 071000 China; ^12^ Research Center for Eco‐Environmental Sciences Chinese Academy of Sciences Beijing 100085 China

**Keywords:** climate change, control measures, crops, human health, mycotoxins

## Abstract

Mycotoxins, a category of fungal secondary metabolites, frequently contaminate food products and pose a severe threat to human health. *Aspergillus*, a genus of fungi, is capable of producing mycotoxins, with aflatoxins (AFs) and ochratoxins being its principal types. *Aspergillus* mycotoxins can contaminate a wide range of crops and their derivatives, such as maize, wheat, rice, minor cereals, and peanuts, thereby threatening food and feed safety. In the paper, the related biosynthesis genes and multifaceted biosynthesis pathways of these mycotoxins are first discussed in detail, and elucidated several global regulators, including growth conditions, oxidative stress, and cell signal. Furthermore, how global shifts in temperature and water availability, driven by climate change (including rising temperatures, increased heavy rainfall frequency, prolonged droughts, and elevated carbon dioxide levels), are key determinants of *Aspergillus* proliferation and mycotoxin production are explored. Finally, to safeguard animal and human health from the detrimental impacts of *Aspergillus* mycotoxins, the effective and convenient analytical techniques and management strategies for the detection and prevention of contamination are analyzed. Overall, this review provides effective detection techniques and promising solutions to the global contamination of food with *Aspergillus* mycotoxins, which is of great significance to ensuring food security and protecting people's lives and health.

## Graphical abstract

Mycotoxins produced by *Aspergillus* contaminate food products and threat to human health. The multifaceted biosynthesis pathways are first discussed and elucidated several global regulators of the mycotoxins in detail. How global shifts in temperature and water availability, driven by climate change, are key determinants of *Aspergillus* proliferation and mycotoxin production are explored. The analytical techniques and management strategies for the detection and prevention of contamination are analyzed.

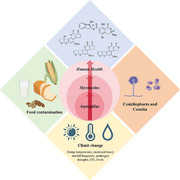

## 1 Introduction

*Aspergillus* is a widespread filamentous ascomycete species prevalent in the natural environment and can exert detrimental effects. For example, *Aspergillus niger* acts as a pathogen for plants as well as an opportunistic pathogen in humans. In human beings, *A. niger* is associated with lung infections, particularly in individuals with compromised immune systems.^[^ ^1^ ^]^ *Aspergillus fumigatus* is the most common airborne fungal pathogen in developed countries and can lead to invasive aspergillosis (IA), which is often fatal in immune‐compromised patients.^[^ ^2^ ^]^ Furthermore, *Aspergillus flavus* is a type of soil fungus that can infect and contaminate pre‐harvest seed crops as well as posted crops, with the carcinogenic secondary metabolite, aflatoxin.^[^ ^3^ ^]^

Mycotoxins pose a substantial threat to both agriculture and foodstuff safety, with that, human health. To date, scientists have identified and characterized over 400 mycotoxins. These substances are highly persistent and continue to exist throughout the food chain. Mycotoxins generally exhibit types of toxicity: short‐term, long‐term of causing genetic mutations and developmental abnormalities. The most frequently noted consequence of short‐term exposure to mycotoxins is the decline in liver or kidney function, which can be deadly in severe instances. For many mycotoxins, the primary long‐term consequence is the development of cancer, particularly affecting the liver.

**Figure 1:**
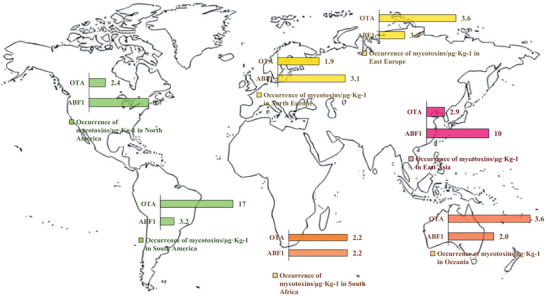
The Occurrence of *Aspergillus* Mycotoxins in Major Regions Worldwide. The legend denotes the median of positive samples for each mycotoxin in each region. The data was sourced from Gruber‐Dorninger et al.^[^ ^210^ ^]^

Mycotoxins are micromolecular metabolites which are produced by fungi, and frequently contaminate food supplies, posing a significant threat to safety. Mycotoxins typically do not play a role in the normal metabolic processes related to fungal growth. However, numerous reports indicate that mycotoxigenic fungi employ various substances to bolster their defensive strategies against other organisms, including microbes, plants, and animals, within their environment. Mycotoxins usually present a variety of chemical structures, which can include straightforward heterocyclic rings with molecular weights starting at 50 Da, as well as intricate arrangements of six to eight‐membered rings with total molecular weights surpassing 500 Da. These compounds demonstrate a broad spectrum of structural arrangements, from the most rudimentary to highly sophisticated molecular configurations.^[^ ^4^ ^]^ As major mycotoxins, aflatoxins and ochratoxin A (OTA), stemming from the prevalent toxigenic fungal genus *Aspergillus*, are two paramount mycotoxins with significant implications for agriculture, economics, and the realm of public health. *Aspergillus* species are known as plant pathogens or commensals and are frequently found in association with foodstuffs during the processes of drying and storage. The contamination of grains with AFs reduces their worth for animal feed and has been related with increased death rates in livestock.^[^ ^5^ ^]^ The OTA, one of the most critical mycotoxins, is significant given its toxic properties and global prevalence, being produced by various species of *Aspergillus*. The two primary *Aspergillus* mycotoxins have already occurred in some major regions worldwide for years (**Figure** **1**). Except for this, fumonisins (FUMs) represent a family of elongated amino polyalcohols, within which FB1 predominates in crop and stands as the most noxious member of the entire cohort.^[^ ^6^ ^]^ Furthermore, patulin (PAT) is responsible for a prevalent post‐harvest disease known as blue mold rot, which affects apples, pears, grapes, and various other fruits.^[^ ^7^ ^]^

The fungi frequently organize the genes responsible for synthesizing secondary metabolites into clusters, facilitating the coordinated activation and regulation of their transcription. In this review, we explore the primary categories of mycotoxins generated by *Aspergillus*, discussing their impact on food safety and human health. Subsequently, the biosynthetic and regulatory pathways of *Aspergillus* mycotoxins were elucidated. Finally, we reviewed the detection technologies and management strategies pertaining to *Aspergillus* mycotoxins contamination. We aim to focus greater attention on the interaction between climate change and the prevention of *Aspergillus* mycotoxins in the future. Meanwhile, we hope the review will provide more helpful information on the prevention of *Aspergillus* mycotoxins.

## 2 Main Mycotoxins in *Aspergillus*

### 2.1 Aflatoxins (AFs)

AFs represent a distinct category of mycotoxins that are predominantly synthesized by toxigenic species of the genus *Aspergillus*, notably *A. flavus* and *A. parasiticus*.^[^ ^8^ ^]^ These compounds exhibit carcinogenic, immunosuppressive, genotoxic, and mutagenic properties.^[^ ^9^ ^]^ AFs have been confirmd that they have acute and chronic toxicological effects in animals and humans, resulting in significant hepatic damage, liver cirrhosis, tumor formation, and teratogenic consequences.

**Figure 2:**
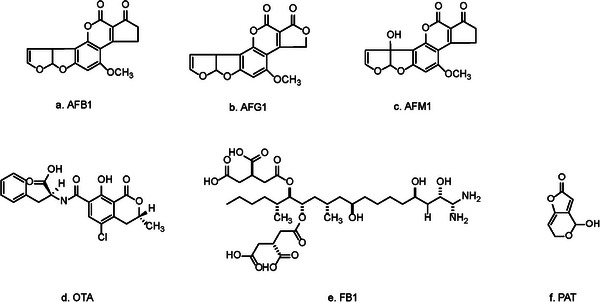
Several main structures of mycotoxins in *Aspergillus*.

AFs exhibit analogous core structures that are derived from dihydrofuran coumarin.^[^ ^10^ ^]^ About twenty‐six types of AFs were discovered up to now [Figure S1A and Table S1, Supporting Information). Given the green or blue fluorescence under UV light, the AFs could be classified into three main groups: aflatoxin B (e. g. aflatoxin B1, AFB1), aflatoxin G (e. g. aflatoxin G1, AFG1), and aflatoxin M (e. g. aflatoxin M1, AFM1) (**Figure** **2**a–c). AFs are the result of a multifaceted biosynthetic pathway that encompasses a minimum of 27 enzymatic reactions.^[^ ^11^, ^12^, ^13^ ^]^ In *Aspergillus*, the AF biosynthesis cluster includes 30 genes and 80 Kb bases (Figure S1B, Supporting Information). 19 core enzymes to synthesize sterigmatocystin/AFs, 2 biosynthesis regulators, 1 major facilitator superfamily transporter, 1 poliketide synthase, 4 anzyme to synthesize aflatoxins, and 3 enzymes to synthesize G‐type afiatoxins are contained in the cluster.^[^ ^14^ ^]^ The detailed biosynthesis process were shown in Figure S1C (Supporting Information).

### 2.2 Ochratoxins

Ochratoxins are another mycotoxin after aflatoxins that has attracted wide attention in the world. Ochratoxins are mainly produced by *Aspergillus* and *Penicillium* genera, and can seriously pollute food and agricultural products (Figure S1A and Table S2, Supporting Information). Among the fungi, OTA (Figure 2d) is the most toxic, the most widely distributed, and the highest toxin production.

OTA is classified as an acute nephrotoxin, with the oral lethal dose for 50% of the population (LD_50_) documented at 20 mg kg^−1^ in juvenile rats.^[^ ^15^ ^]^ It was originally produced by *A. ochraceus*, *A.carbonarius*, *A. niger*, and *P. verrucosum*. OTA is among the most prevalent mycotoxins found in the diets of both humans and animals, and prolonged exposure has been linked to significant renal damage.^[^ ^16^ ^]^ It has been reported that OTA has immunosuppressive, embryonic, and probably carcinogenic effects in total. OTA comprises a para‐chlorophenolic component that is associated with a dihydroisocoumarin group, which is connected to L‐phenylalanine.^[^ ^17^ ^]^ The biosynthesis genomic clusters typically includes 5 conservative genes, and they are named *pks* (*otaA*) (polyketide synthase), *nrps* (*otaB*) (nonribosomal peptide synthetase), *p450* (*otaC*) (P450 monooxygenases), *hal* (*otaD*) (radH flavin‐dependent halogenase), and *bzip* (*otaR1*) [bZIP transcription factor (TF)]^[^ ^18^ ^]^ (Figure S1B, Supporting Information). The AcOTAPKS facilitates the synthesis of the isocoumarin moiety in the early phases of biosynthesis. This process commences with the precursors acetate and malonate, ultimately leading to the development of the distinctive pentaketide framework of the OTA molecule (Figure S1D, Supporting Information). All in all, a clear biosynthesis pathway of OTA could provide us more information for its controlling in the future.

### 2.3 Fumonisins (FUMs)

The FUMs are a significant group of mycotoxins produced primarily by the cereal pathogen, *A. niger*. A number of other FUMs are mainly produced by *Fusarium* spp (Figure S1A and Table S3, Supporting Information).^[^ ^19^ ^]^ FUMs are composed of a 20‐carbon aliphatic chain that features two hydrophilic side chains linked by ester bonds, exhibiting structural similarities to sphingosine, which is a crucial phospholipid found in cellular membranes.^[^ ^15^ ^]^ More than 28 homologues including fumonisin B1 (FB1) (Figure 2e), have been discovered and more are likely to be found up to now. In *A. niger* genome, the biosynthesis of FUM was achieved by a 37‐kb gene cluster and 11 related genes, which were named namely *fum1* (polyketide synthase), *fum3, fum6*, and *fum15* (hydroxylase), *fum7* (dehydrogenase), *fum8* (aminotransferase), *fum10* (acyl‐CoA synthase), *fum13* (carbonyl reductase), *fum14* (condensation‐domain protein), *fum19* (ABC transporter), and *fum21* (transcription factor) genes^[^ ^20^, ^21^ ^]^ (Figure S1B, Supporting Information). The biology pathway was started with the conformation of 10,14‐dimethyloctadecanoic acid (Figure S1E, Supporting Information). The synthesis of fumonisin is contingent upon the *FUM1* gene, which encodes for an enzyme complex referred to as polyketide synthase. This complex facilitates the initial step in the biosynthetic pathway of fumonisin. It is noteworthy that certain species of *Aspergillus* are capable of producing fumonisin variants B2, B4, and B6; however, they do not synthesize the most toxic variant, FB1.^[^ ^22^ ^]^

### 2.4 Patulin (PAT)

PAT, is a polyketide lactone [4‐hydrox y‐4H‐furo (3,2‐c) pyran‐2(6H)‐one] produced primarily by *Aspergillus*, *Penicillium* and *Byssochlamys* species^[^ ^23^ ^]^ (Figure 2f). In *Aspergillus*, the PAT producing species includes *A. clavatus*, *A. giganteus* and *A. longivesica*.^[^ ^24^ ^]^ PAT was observed to exhibit substantial growth on fruit and vegetables such as apples, peaches and apricots.^[^ ^25^ ^]^ A substantial body of research has demonstrated that acute exposure to PAT leads to a range of adverse effects, including the observed effects such as ulceration, agitation, convulsions, edema, vomiting, and damage to DNA within the brain, kidneys, and liver.^[^ ^26^ ^]^

The biosynthetic cluster responsible for the production of PAT has been preliminarily characterized in *Aspergillus clavatus*. The process involves 10 enzymatic steps and is associated with a cluster of 15 genes that have been identified and characterized in *A. clavatus* (Figure S1B, Supporting Information). Eleven genes, including *PatK*, *PatH*, *PatI*, and *PatN*, are accountable for the enzymatic processes involved in the production of PAT, respectively.^[^ ^27^ ^]^ The biosynthetic pathway of PAT commences with the involvement of acetyl coenzyme A (CoA) and malonyl CoA, and finally produces PAT^[^ ^25^ ^]^ (Figure S1F, Supporting Information).

Mycotoxins are wide‐spread and of concern in food/feed safety, human life, and soil environment with decades years. An examination of the interactions between the mycotoxin cluster and the regulatory factors associated with biosynthesis may provide insights into novel strategies aimed at mitigating mycotoxin production. This could be achieved by specifically targeting the genes or proteins that are involved in the upstream regulation of the mycotoxin cluster.

## 3 Health Effects of *Aspergillus* Mycotoxins

**Figure 3:**
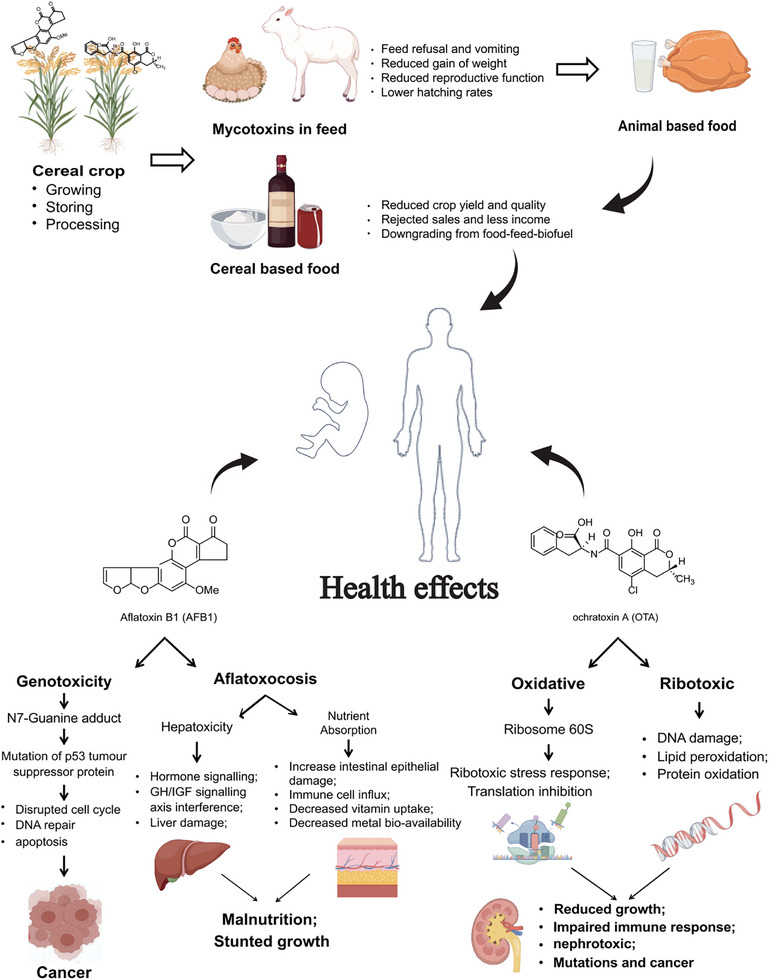
Transmission and health effects of *Aspergillus* mycotoxins. Mycotoxins might come from different stages of food stuffs such as crops, animal‐derived meat product, and cheese, and transmits by the food chain to human beings. Mechanisms of AFB1 induced cancer, malnutrition, and stunted growth, and OTA induced impairment of reduced growth, impaired immune response, nephrotoxic, mutations, and cancer.

Toxigenic fungi, such as species of *Aspergillus*, are known to contaminate cereal crops with various deleterious mycotoxins. The mycotoxins could threat not only food security but also the health of animals and humans (**Figure** **3**).

### 3.1 Crop and Feed Insecurity

Fungi represent significant pathogens affecting crops as well as being responsible for the spoilage of food products. Opportunistic pathogens, including *A. flavus* and *A. parasiticus*, have the potential to contaminate cereals both in agricultural settings and during storage processes. *Aspergillus* have broad host ranges that include important cereal crops such as maize (lead to root, stem, and ear rot), wheat (lead to wheat scab), rice (lead to ear rot), oat, and barley. For example, AFs were detected in all food cereals over the last decade (2010–2020), with 100% in maize, 88.23% in wheat, 78.57% in rice, and 14.28% in oats being isolated.^[^ ^28^ ^]^ OTA is often found in food and crops such as coffee, beer, wine, corn, wheat, oats, and vegetables.^[^ ^29^ ^]^ The agricultural commodity contamination takes place at various stages, including pre‐harvest, harvest, and post‐harvest phases, which encompass processing, packaging, storage, and transportation. Furthermore, FB1 is predominantly produced by fungi present in the crop during its growth phase prior to harvest, resulting in challenges associated with the management of these fungi.^[^ ^30^ ^]^ It mainly contaminates corn and its products. It has been reported that 404–420 feeds were contaminated in feed tests in our country, with the highest being 6568 µg Kg^−1^.^[^ ^31^ ^]^ At present, the FB_1_ in wheat products in not at high levels and is relatively less harmful to human beings. By the way, the presence of PAT in food items, particularly in fruits and vegetables, poses a considerable challenge for the food industry. The European Union has established maximum allowable concentrations of PAT in various apple‐derived products, specifying a threshold of 50 µg kg^−1^ for apple juice, 25 µg kg^−1^ for solid apple products, and 10 µg kg^−1^ for products intended for infants and young children.^[^ ^32^ ^]^

The *Aspergillus* mycotoxins and their masked derivatives have nonnegligible health implications across the food and feed supply chains (Figure 3).

### 3.2 Effects on Animal and Human Health

AFB1 is considered as a hazardous substance for both animals and human beings, as it is associated with heightened DNA mutations, cellular apoptosis, and the onset of liver cancer. It is worth noticing that malnutrition could also occur when AFB1 consumption results in hepatoxicity. AFs adversely affect the poultry industry by prolonging the maturation process of birds, impeding weight gain, diminishing egg production, and compromising overall quality.^[^ ^33^ ^]^ Besides, egg quality is predominantly impacted via AFs. Except that, it is of significant concern that AFM1 may be present in the breast milk of both animals and humans, as milk contaminated with aflatoxins cannot be effectively deactivated through heat or pasteurization processes. Followed, milk contaminated with AFM1 may pose significant health risks to children, potentially leading to impaired growth in infants and heightened vulnerability to infectious diseases.^[^ ^34^ ^]^ In both animal and human livers, the consumption of food or feed contaminated with AFB1 leads to the enzymatic conversion of AFB1 into its hydroxylated derivative, AFM1, along with other metabolites. This process is facilitated by microsomal mixed‐function oxidase enzymes, specifically CYP1A2 and CYP3A4, which may impart carcinogenic characteristics to AFB1.

OTA was classified as a Group 2B carcinogen by the International Agency for Research on Cancer.^[^ ^35^ ^]^ Studies demonstrated that OTA exhibits nephrotoxic, hepatotoxic, teratogenic, carcinogenic, and immunosuppressive properties.^[^ ^36^ ^]^ Pigs are the most susceptible to nephrotoxicity. Ochratoxins present in turkeys and chickens have the potential to induce nephrotoxicity, result in weight loss, decrease egg production, and adversely affect eggshell quality.^[^ ^37^ ^]^ It has been established that OTA is implicated in human Balkan endemic nephropathy (BEN), as well as in renal failure and the development of tumors.^[^ ^38^ ^]^ Research has also demonstrated that OTA functions as an inhibitor of Nrf2, thereby inflicting cellular damage through the induction of physiological oxidative stress. This oxidative stress subsequently results in DNA damage and the occurrence of mutations.^[^ ^39^ ^]^ FB1 has immunotoxicity and organ toxicity. The research demonstrated that the intragastric administration of FB1 at a dosage of 80 mg kg^−1^ body weight to mice over a 14‐day period resulted in a reduction in spleen weight and induced 12.9% apoptosis in thymocytes.^[^ ^40^ ^]^ FB1 has the potential to suppress the non‐specific immune system in pigs, leading to a reduction in macrophage capacity and an exacerbation of pathogen infections. In humans, lymphocyte survival was controlled by 3.5% after one day of exposure to concentrations of 5 µg mL^−1^ of FB1, and 11.3% of 20 µg mL^−1^, respectively.^[^ ^41^ ^]^ A series of studies have demonstrated that FUMs demonstrate toxicological effects on multiple organ systems, including the liver, lungs, kidneys, heart, and intestines, in various animal species.^[^ ^42^ ^]^ It was worth noticing that FUMs could cause damage to brain. In Kenya, FB1 may induce cancer.^[^ ^43^ ^]^ Except that, the impact of FB1 on the reproductive system mainly involves reproductive issues and underdeveloped fetuses in specific animal species.^[^ ^44^ ^]^

Humans are exposed to multiple mycotoxins through various routes.^[^ ^45^ ^]^ It is important to note that the occurrence and co‐occurrence of multiple mycotoxins are frequently present in commercially available food products. Exposure to fungi via food is virtually unavoidable, as these organisms possess the ability to endure environmental conditions that would generally be detrimental to other microorganisms.^[^ ^46^ ^]^ Daily dietary exposure to mycotoxins, as well as exposure to combinations of mycotoxins, is a frequent occurrence. Andrews‐Trevino et al.^[^ ^47^ ^]^ found that exposure to multiple mycotoxins was prevalent among young children. Many mycotoxins independently contribute to various manifestations of negative growth in children. AFB1 exhibited a negatively correlation with length, weight, and head circumference, whereas deoxynivalenol (DON) demonstrated a negative correlation specifically with head circumference. Furthermore, Kyei et al.^[^ ^48^ ^]^ reported that exposure to multiple mycotoxins during pregnancy is widespread in the rural communities investigated, posing a serious health risk for both mothers and infants. Current mycotoxin regulations primarily rely on toxicological data from individual mycotoxin exposures and do not consider the potential combined effects of simultaneous exposure to multiple mycotoxins or the influence of interacting risk factors.^[^ ^49^ ^]^

Meanwhile, the prolonged presence of low levels of mycotoxins under specific environments, such as workplaces or living spaces, along with repeated exposure to contamination, could pose a significant threat to human health. This exposure is particularly relevant in agricultural settings and the food industry.^[^ ^50^ ^]^ Work environments characterized by ventilation, inadequate protective clothing, and insufficient equipment increase the risk of contamination for workers.^[^ ^51^ ^]^ To date, exposure assessment remains a challenging endeavor, as it requires comprehensive data to effectively research the effects of mycotoxins on human health and draw sound conclusions.

To this day, the number of immune defense mechanisms against the analyzed mycotoxins in mammals and humans remains quite limited. To mitigate the risk of developing health issues, many identified mycotoxins are regulated by European legislation. However, several ones are still considered emerging concerns, and their effects on human health are not well understood. These issues require urgent attention in the future.

## 4 Regulators of *Aspergillus* and Mycotoxin

### 4.1 Cluster‐Specific Regulators

**Figure 4:**
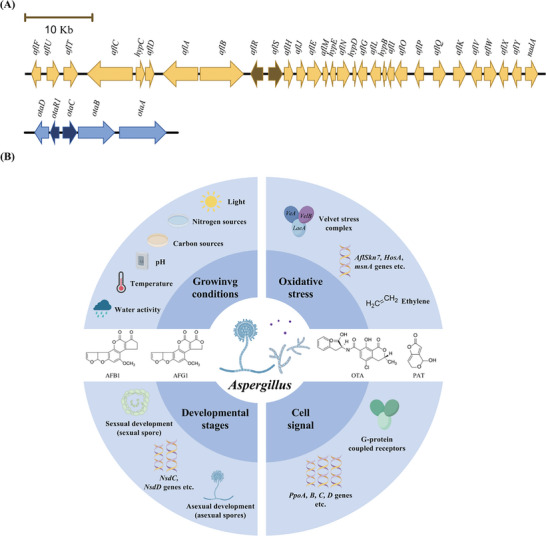
Regulators of *Aspergillus* mycotoxins. A) Cluster‐specific regulators on aflatoxins and ochratoxins biosynthetic gene clusters. The related four genes are marked by darker colors. B) Global regulation factors related with *Aspergillus* mycotoxins. Growing conditions, developmental stage, oxidative stress, and cell signal and their regulators are listed in the picture.

Gene clusters involved in fungal secondary metabolism often contain one or more transcription factors that are crucial for the expression of biosynthetic genes.^[^ ^52^ ^]^ Molecular studies have demonstrated that the synthesis of AFs is regulated by complex cascades of transcriptional and post‐transcriptional processes.^[^ ^53^ ^]^ The AFs biosynthesis cluster genes expression is regulated by two specific genes: *aflR* and *aflS* ^[^ ^54^ ^]^ (**Figure** **4**A).

AflR is a zinc cluster Zn(II)₂Cys₆ transcription factor, which belongs to Gal4‐type family.^[^ ^55^ ^]^ Such transcription factors can bind to DNA through a DNA‐binding domain, which is a crucial component in transcriptional and translational processes.^[^ ^56^ ^]^ *AflR* could recognize the palindromic pattern 5′‐TCGNNNNNCGA‐3′, 5‘‐TTAGGCCTAA‐3′ and 5′‐TCGCAGCCCGG‐3′.^[^ ^57^ ^]^ The overexpression of *aflR* could laed to highly increasing in AF production in *A. flavus*.^[^ ^58^ ^]^ At the same time, Δ*aflR* gene in *A. parasiticus* could decrease AF pathway genes and mycotoxins.^[^ ^59^ ^]^

The *aflS* gene located in the AF biosynthetic cluster and could also regulated by *aflR*.^[^ ^60^ ^]^ The knockout of *aflS* in *A. parasiticus* resulted in reduction of *AFB1* genes.^[^ ^54^ ^]^ Meanwhile, overexpression of *aflS* resulted in higher production of AFB1, along with the increasing in *aflC* and *aflD* expression in *A. flavus*.^[^ ^61^ ^]^ Previous studies proposed that the dimer‐complex (including 4 AflS for 1 AflR) can clarify the specific sites in *aflC* and *aflD*, and increase their transcription.^[^ ^61^, ^62^ ^]^

In *A. carbonarius*, the *AcOTAp450* and *AcOTAbZIP* genes encode a cytochrome P450 monooxygenase and a bZIP transcription factor, respectively, both of which have homologous genes in the OTA cluster^[^ ^63^ ^]^ (Figure 4A). The P450 monooxygenase involved in OTA biosynthesis is likely responsible for an oxidative step in the formation of OTβ within the biosynthetic pathway. The bZIP transcription factor present in the gene cluster may play a significant role in regulating OTA gene expression. Additionally, *otaE* [flavin adenine dinucleotide (FAD)‐dependent oxidoreductase] and *otaR2* (zinc finger DNA‐binding protein) have been identified adjacent to the core cluster in *A. ochraceus* and in proximity to the otaA gene.^[^ ^64^ ^]^ The coding sequence length of the o*taR2* gene is conserved in nearly all the aforementioned species such as *Aspergillus sclerotioniger*, *Aspergillus carbonarius*, and *Aspergillus niger*. In contrast to the other mycotoxins, the biosynthesis of OTA is not completely clear until recently.

Another cluster‐specific regulators also have been reported on fumonisins. *Fum21*, which is located adjacent to *fum1* and reported in *F. verticillioides* first, was described as a transcriptional regulator of fumonisins biosynthesis cluster.^[^ ^21^ ^]^ The protein FUM21 contains a Zn(II)2Cys6 DNA‐binding domain, indicating its potential role in transcriptional regulation. The deletion mutants of *fum21* would lead to decreasing of biosynthetic genes such as *fum1* and *fum8*. The results suggested that the *fum21*could evidently regulated the FUMs biosynthesis.^[^ ^65^ ^]^

In *A. clavatus*, the PAT biosynthesis genes are located within a 40‐Kb cluster that consists of ≈15 genes, including 3 transporter genes: 1 putative ATP‐binding cassette (ABC) transporter, 1 major facilitator superfamily (MFS) transporter, and 1 acetate transporter.^[^ ^66^ ^]^ The transcription of the putative ABC transporter gene in PAT biosynthesis cluster is up‐regulated under patulin‐permissive conditions. PatL is a putative specific transcription factor associated with the PAT pathway. The deletion of PatL significantly impairs the expression of all genes within the PAT cluster and results to the cessation of patulin production.^[^ ^67^ ^]^ However, the specific PatL‐binding site on the PAT cluster is not clear. Although the chemical structures of PATs have been reported, the roles of all BGC genes and specific regulators are still unclear up to now.

### 4.2 Global Regulation

Global regulatory factors, in this context, pertain to genes that are not part of the mycotoxin gene clusters. But they have been shown to be associated with the production of mycotoxins. Mycotoxins biosynthesis could be repressed by environmental stimuli, such as nutrient sources. A substantial body of research has demonstrated that the production of mycotoxins, particularly AFB1, is primarily influenced by environmental factors.^[^ ^68^, ^69^ ^]^ In this section, we described the recent researches demonstrated to link the global regulatory factors and mycotoxins production (Figure 4B).

#### 4.2.1 Growing Conditions and Developmental Stage

The conditions under which fungi are cultivated significantly impact the production of secondary metabolites. For instance, previous research has demonstrated the effects of water activity and temperature on the growth and aflatoxin production of *A. flavus* and *A. parasiticus*. Additionally, several environmental parameters, including carbon sources, nutrient availability, pH levels, and light exposure, have been shown to directly influence mycotoxin production. Furthermore, the involvement of specific genes in this phenomenon has been emphasized in the literature.^[^ ^71^ ^]^ Some related genes are listed in **Table** **1**.

**Table 1:** The regulation factors related to the growing conditions and developmental stages of *Aspergillus* and mycotoxins production.

Main factors	Species	Regulation factors/genes	Mutant	Phenotype or gene coding function
Carbon sources	*A. flavus*, *A. nidulans* and *A. parasiticus*	Simple sugars compared with D‐glucal used as carbon sources in the culture	–	Higher levels of AF production.
	*A. flavus* and *A. parasiticus*	*CreA* gene	Gene deletion	Growth defect on complete medium, conidia production, impaired ability to colonize hosts and produced insignificant amount of AF.
		*CreB* gene	Gene deletion	Cys_2_His_2_ Zinc finger ubiquitin processing protease; produced insignificant amount of AF.
		*CreC* gene	Gene deletion	Cys_2_His_2_ Zinc finger ubiquitin processing protease; produced insignificant amount of AF.
		*alcR* gene	–	Specific transcription factor
		*aldA* gene	–	Aldehyde dehydrogenase
		*alcA* gene	–	(Same as above)
Nitrogen sources	*A. parasiticus*	*aflC* and *aflD* genes	–	They could expressed on ammonium and peptone media, but could not grow on nitrate sources.
	*A. nidulans*	–	–	The level of sterigmatocystin increased in the nitrate medium but not in the ammonium medium.
	*A. flavus*	*areA* gene	Loss of function or Overexpression *areA*	Increased/decreased levels of AFs on nitrogen source mediums.
		*meaB* gene	Overexpression	Could not produce AFs; regulatory protein.
		*nmrA* gene	–	Repressive nitrogen
		*niiA* gene	–	Nitrite reductase
		*niaD* gene	–	Nitrite reductase
pH	*A. flavus*	*pacC* gene	–	High pH leads to decrease levels of AF, but low pH (<4.0) resulted in increasing of AF on nitrate‐based medium.
	*A. parasiticus*	*pacC* gene	–	Low pH (<6.0) result to B‐type AF production, but high pH result to G‐type production.
		*aflM* gene	–	The level of AF production increases on the medium.
	*A. nidulans*	*stcU* (*aflM* homologous gene)	–	The gene expression level is lower in an increased pH medium.
Light	*A. carbonarius, A. niger, A. westerdijkiae*	White light	–	Seems to consistently reduce OTA production.
	*A. steynii*	Yellow and green light	–	Increased OTA production.
	*A. carbonarius*	Dark conditions	–	The production of OTA was promoted.
	*A. carbonarius*	Red light	–	Increased OTA production.
	*A. ochraceus*	UV light treatment		Conidia formation, mycelial growth, and OTA production are affected.
	*Aspergilli* spp.	*VeA* gene	Knockout‐deletion *VeA*	Down‐regulation of the NRPS encoding genes and reduction of OTA produced; global regulator.
		*laeA* gene	Knockout‐deletion *laeA*	Reduction of OTA produced; Putative methyltransferase.
		*llmF* gene	–	LaeA‐like methyltransferase
		*velB* gene	–	Vea‐LaeA‐VelB trimeric (velvet) complex, response to light signals, secondary metabolism and fungal development; Velvet‐like protein B
		*lreA* gene	–	Blue‐light sensing protein
		*lreB* gene	–	Blue‐light sensing protein
		*fphA* gene	–	Phytochrome‐like red light receptor
Developmental stage	*A. niger*	*NsdC* gene	Knockout‐deletion *NsdC*	Decreased conidium production, loss of sclerotia, radial growth and ochre conidial pigmentation, the speed of AF accumulation much lower than the wild type.
		*NsdD* gene	Knockout‐deletion *NsdD*	Decreased conidium production, loss of sclerotia, decreased radial growth and slightly light green, the speed of AF accumulation much lower than the wild type.
		*Hbx1* gene	Knockout‐deletion target gene	Could nou product conidia, sclerotia, AFB1, AFB2, cyclopiazonic acid and aflatrem.
	*A. flavus*	*BrlA* gene	Knockout‐deletion target gene	Down‐regulation the conidiophore development and *aflC, aflD, aflM* and *aflR* genes.
		*AbaA* gene	Knockout‐deletion target gene	Downregulation the conidiophore development, AFB1 and AFB2.
		*PbsB* gene	Knockout‐deletion target gene	Up‐regulation of *aflR, aflC, aflD, aflK*, and *aflQ* and increased AFB1 production.
		*AfRafA* gene	Loss of function	Suppressed AF cluster expression, reduced mycotoxin synthesis, and impaired conidia and sclerotia development.
		*AfStuA* gene	Loss of function	Aflatoxin biosynthesis, conidia and sclerotia development were fully blocked.
		*Rum1 gene*	Deletion mutants and complementation strains	Inhibit conidiation, increase sclerotia formation, and AF synthesis
	*A. nidulans, A. parasiticus*	*FadA* gene	–	Asexual development, Inhibition of conidiation, aflatoxin biosynthesis and pathogenicity
	*A. clavatus*	*Set1* gene	Deletion of target gene	Inhibit hyphal growth, conidiation, colonization and PAT biosynthesis.

Previously reports have shown that the availability and type of carbon source could influence the production of SMs. Simple sugars, such as sucrose, glucose, fructose, and sorbitol, have been associated with increased levels of AF production in species such as *A. flavus, A. parasiticus*, and *A. nidulans*.^[^ ^72^, ^73^, ^74^ ^]^ D‐glucal as the primary sugar source in culture has been shown to inhibit AF production.^[^ ^75^ ^]^ Carbon catabolic repression may serve as a strategic mechanism employed by *Aspergilli* to conserve energy and regulate carbon catabolism, thereby optimizing the utilization of the most advantageous carbon source.^[^ ^71^ ^]^

In *Aspergillus* species, nitrogen sources has been performed that it could effect mycotoxins production in several pathways and be regulated by the nitrogen metabolite repression mechanism.^[^ ^73^ ^]^ The nitrogen source is intricately associated with AF production, as certain substrates, such as asparagine, ammonium salts, and glutamate, facilitate aflatoxin synthesis, whereas others, including sodium nitrate and tryptophan, do not support this process.^[^ ^14^ ^]^ It has been proposed that nitrate may inhibit the formation of averufin and aflatoxin.^[^ ^76^ ^]^ The nitrogen utilization gene, for example, *areA* from *A. parasiticus* has previously been cloned.^[^ ^77^ ^]^ In the intergenic region located between *aflR* and *aflS*, referred to as *aflJ*, multiple AreA binding motifs have been identified, suggesting that AreA binding may obstruct AflR binding.^[^ ^78^ ^]^ Grootwassink and Gaucher^[^ ^79^ ^]^ have documented that resuspending mycelium in a nitrogen‐free 4% glucose solution promptly activates the pathway enzymes and leads to the production of PAT. Furthermore, the introduction of ammonium ions to cultures that are actively producing PATs results in a rapid decline in secondary metabolism and a concomitant decrease in PAT production.^[^ ^80^ ^]^

Microorganisms are significantly impacted by alterations in the ambient pH of their surroundings. pH represents an extracellular condition that fungal organisms respond to and which influences the production of secondary metabolites. A putative PacC‐binding site in proximity to the *aflR* transcription start site may contribute to the regulation of pH in AF production.^[^ ^81^, ^82^ ^]^ In a peptone medium that does not support aflatoxin production, this site has been demonstrated to inhibit aflatoxin formation. The underlying regulatory mechanism is likely attributed to the *PacC* binding to the site under alkaline, which represses the acid‐expressed gene *aflR* transcripting, thereby reducing AFs production.^[^ ^83^ ^]^ Furthermore, gene deletion and complementation analyses indicate that PacC is essential for mycelial growth, conidiation, and virulence. Furthermore, two novel virulence factors, PeCrt and PeSat, have been identified as being under the regulation of PacC. Summerer^[^ ^84^ ^]^ reported that the culture medium pH could also affect the *idh* gene, which leads to the production of PAT.

Light is crucial in influencing the production of mycotoxins by numerous fungal species, which is threat to food safety. The in vitro capacity of *Aspergillus* species to produce mycotoxins is significantly enhanced under dark conditions.^[^ ^85^ ^]^ The translocation of the VeA transcription factor could be inhibited by light.^[^ ^85^ ^]^ In *A. flavus*, the knockout of *VeA* gene exhibited *aflR* gene, which was a specific transcription factor, as well as the primary genes involved in aflatoxin biosynthesis genes.^[^ ^86^ ^]^ The role of the velvet complex has been documented in some instances in light responsing. It could influence the production of AFs in *A. parasiticus* and *A. flavus*, ^[^ ^87^, ^88^ ^]^ OTA production by *A. carbonarius*, ^[^ ^89^ ^]^ and FUM production by *F. verticillioides*.^[^ ^90^, ^91^ ^]^ The impact of light at varying wavelengths on OTA production has also been documented by numerous research groups. Whereas, in *A. steynii*, yellow and green light could lead to the increasing of OTA, and red light could also lead to increase in *A. carbonarius*.^[^ ^92^, ^93^ ^]^

The secondary metabolism has been reported that they are related with the sporulation and sclerotial formation.^[^ ^73^ ^]^ Certain compounds that have the potential to inhibit sporulation in *A. parasiticus* also demonstrate the ability to inhibit aflatoxin production.^[^ ^94^ ^]^ Furthermore, substances that suppress polyamine biosynthesis in both *A. parasiticus* and *A. nidulans* also impede sporulation as well as the production of aflatoxin/ST.^[^ ^95^, ^96^ ^]^ The deletion of the *Set1* protein resulted in PAT biosynthesis.^[^ ^97^ ^]^

#### 4.2.2 Oxidative Stress

Oxidative stress is defined as a state in which there is a disruption in the equilibrium between the generation of reactive oxygen species (ROS) and the cellular mechanisms responsible for antioxidant defense.^[^ ^98^ ^]^ Fungi respond to oxidative stress through the synthesis of antioxidant molecules that mitigate the effects of oxidants. Oxidative stress and mycotoxins biosynthesis are related in *Aspergillus*.^[^ ^99^, ^100^, ^101^ ^]^ Gallic acid has been shown to reduce the expression of AF biosynthesis genes. However, it does not affect gene regulators such as *aflR*. Recent studies have confirmed that, in *A. ochraceus*, tert‐butyl hydroperoxide or carbon tetrachloride increased the production of OTA. Similarly, oxidative stress induced by FB1 resulted in cytotoxicity and a reduction in cellular activity.^[^ ^102^ ^]^ Some related genes are listed in **Table** **2**.

**Table 2:** The regulation factors related to the oxidative stress of *Aspergillus* and mycotoxins production.

Species	Regulation factors/genes	Mutant	Phenotype and gene function
*Aspergillus* spp.	Use of ethylene or BHA	–	Down‐regulate biosynthetic genes and inhibit mycotoxin production.
*A. flavus*	*AflSkn7* gene	Deletion of the target gene	Especially sensitive to oxidative stress, exhibited partially defective conidial formation and decreased aflatoxin biosynthesis
	*HosA* gene	Deletion of the target gene	Reduced aerial hyphae, significantly decreased colony growth and increased tolerance to oxidative stress generated by hydrogen peroxide (H_2_O_2_).
	*acyA* gene	Deletion of the target gene	Down‐regulated *aflR* and *aflO* genes and could not produce AF; Adenylate Cyclase.
	*PbsB* gene	–	Increase AFB1 production; MAP kinase kinase.
	*msnA* gene	Deletion of the target gene	Higher levels of ROS and increase mycotoxins; transcription factor.
	*Ap1* gene	Deletion of the target gene	Down‐regulate *aflM* and *aflP*, up‐regulate *aflR* and reducing AFs production; bZIP transcription factor.
*A. parasiticus*	Increased oxidative stress	–	Aflatoxin production.
	*ApyapA* gene	Knockout	Increase AFs production.
*A. ochraceus*	The presence of t‐butyl hydroperoxide or carbon tetrachloride	–	Triggered OTA production.
	*Aoyap1* gene	Knockout	Increased OTA and the conidia formation was significantly reduced.
	*Lox* gene	–	Inhibited OTA production.

#### 4.2.3 Cell Signal

Cell signaling mechanisms in fungal cells may facilitate their adaptation to environmental stressors by initiating prompt signal transduction pathways within the cellular structure.^[^ ^103^ ^]^ The Gα subunit FadA is part of an adenylate cyclase/cAMP/protein kinase A (PkaA) pathway. The pathway could control the secondary metabolism production.^[^ ^55^, ^104^ ^]^ OTA leads to sustained activation of PI3K/AKT and MAPK/ERK1‐2 pathways had previously demonstrated.^[^ ^104^ ^]^ Except that, FB1 has the capacity to enhance the expression of mRNA associated with the TNF signaling pathway in porcine kidney cells (PK‐15). Furthermore, tumor necrosis factor α (TNFα) is identified as a critical agent responsible for inducing toxicity.^[^ ^105^ ^]^ Likewise, Set1, which involves in the biosynthesis of PAT, positively regulates the expression of core genes in *β*‐1,3‐glucan biosynthesis, thereby modulating oxidative stress responses.

Numerous genes implicated in various cellular functions have been identified as capable of directly or indirectly modulating the biosynthesis of mycotoxins in *Aspergillus* species.^[^ ^103^ ^]^ Previous research has established connections between various fungal developmental processes and G‐protein signaling pathways. The Gα subunit FadA is a component of the adenylate cyclase/cAMP/protein kinase A (PkaA) pathway, which regulates the production of secondary metabolites through both transcriptional and posttranscriptional modulation of aflR in *Aspergillus* species such as *A. parasiticus*, and *A. flavus*.^[^ ^55^, ^104^ ^]^ Specifically, in activates the through pathway, in part, through the activation of the activation.^[^ ^104^ ^]^ Additionally, FB1 has been shown of TNF signaling pathway‐related to the tumor necrosis factor porcine pathway (PK‐15) cells with TNFα, is a key substance being toxicity.^[^ ^105^ ^]^ Furthermore, biosynthesis is involved in the positive regulation of PAT in β‐1,3‐glucan biosynthesis. It is also associated with the scavenging of reactive oxygen species, thereby contributing to the integrity of cellular processes and responses to oxidative stress and other stressors.^[^ ^106^ ^]^ Some related genes are listed in **Table** **3**.

**Table 3:** The regulation factors related to the cell signal of *Aspergillus* and mycotoxins production.

Species	Regulation factors/genes	Mutant	Phenotype
*A. flavus*	*GprK* gene	Deletion	Increased AF production
	*GprA* gene	Deletion	Increased AF production
	*Lox* gene	Disrupted	Increased AF productionon in maize and peanut seeds.
	*PpoA, B, C, D* gene	Disrupted	Increased AF productionon in maize and peanut seeds.
*A. nidulans*	*PpoA* gene	Deletion	Unable to produce mycotonxins.
	*PpoB* gene	Deletion	Unable to produce mycotonxins.
	*PpoC* gene	Deletion	Unable to produce mycotonxins.
	*RasA* gene	–	Control the *aflR* activity.
	*GprH* gene	–	Negative regulators of mycotonxins biosynthesis.
	*GprM* gene	–	Negative regulators of mycotonxins biosynthesis.

Numerous recent studies have shown an interplay between the genes and factors responsible for responding to environmental stimuli and those within the mycotoxin biosynthesis gene clusters. Considering the intricate nature of the pathways involved and their numerous interconnections, the mechanisms underlying such interactions are frequently not fully comprehended. Comprehending the effects of global environmental cues on cluster‐specific regulators and pinpointing the genes implicated is an ambitious objective. Further regulatory factors should be explored to better control contamination by *Aspergillus* and mycotoxins in the next phase. We advocate for the application of untargeted methodologies, including RNA‐seq and microarray techniques, to identify the inhibition of mycotoxin biosynthesis clusters genes. It has the potential to enable the identification of novel targets for controlling mycotoxin production, thereby ensuring food safety through eco‐friendly and sustainable methods.

### 4.3 Climate Change and Macro Environment

Based on the Sixth Assessment Report of the Intergovernmental Panel on Climate Change, the climate is experiencing significant global changes as a direct consequence of human activities.^[^ ^107^ ^]^ The speed of Carbon dioxide emissions into the atmosphere contributed to an increase of the Earth's average surface temperature of ≈1.18 °C since the late 100 years. According to scientific evidence, climate change (including encompassing temperature variations, water availability, light quality, quantity, etc) is projected to persist, with temperature rises estimated between 1.4 and 5.5 °C over the next ten decades. Additionally, atmospheric concentrations of CO_2_ are expected to double or triple, increasing from 350–400 ppb to 800–1200 ppb parts per billion within next 25 to 50 years.^[^ ^108^ ^]^ The global warming resulting from climate change, especially the heightened frequency and severity of heat waves, has been associated with effects on the growth, proliferation, and production of mycotoxins by mycotoxigenic fungi.^[^ ^109^ ^]^

**Figure 5:**
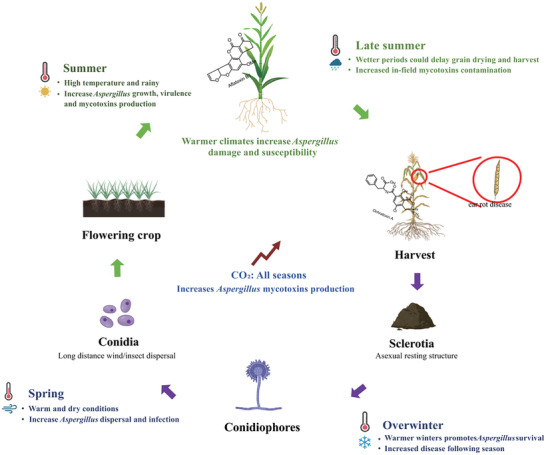
Climate change impacts on risk of cereal contamination by *Aspergillus* influence of climatic conditions on life process. Climate change is anticipated to result in warmer and drier conditions during the spring season. Conversely, summer is projected to experience higher temperatures accompanied by increased rainfall, which is conducive to the growth of *Aspergillus* species. Throughout all seasons, elevated levels of carbon dioxide are correlated with heightened mycotoxin contamination. Consequently, projections related to climate change may indicate a potential rise in mycotoxigenic fungal contamination of cereal crops.

Given the profound impact of mycotoxins, there has been a recent surge in interest regarding the influence of environmental factors.^[^ ^109^ ^]^ Interacting climate abiotic factors, particularly temperature, and possibly atmospheric CO_2_ levels, significantly affect the infection of food crops by mycotoxigenic fungi.^[^ ^110^ ^]^ Although AFs and ochratoxins can be detected worldwide, the varying optimal production temperatures and water activity (a_w_) values of *Aspergillus* fungi contribute to their uneven distribution (**Table** **4**). Gaining a precise understanding of the exact impact of future climate change on fungal ecology remains a formidable challenge (**Figure** **5**). Numerous predictions previously on the influence of climate change on fungi and mycotoxins only consider limited interactions.

**Table 4:** The optimal temperature and aw of *Aspergillus* mycotoxin production.

Species	Mycotoxins	Temperature [°C]	a_w_	Time [days]	Concentration [µg g^−1^]
*A.flavus*	AFB1	25–34	0.90–0.99	7–9	18.0–76.0
	AFB2	15–34		3–21	0.200–167
	AFG1	15–32		3–9	0.600–458
	AFG2	18–32		3–9	3.00–560
*A.carbonarius*	OTA	20–28	0.94–0.98	15	0.122–2.00

The optimal temperature and a_w_ of AFB1, AFB2, AFG1, AFG2 and OTA production were detected on *Aspergillus* infected maize grain.

#### 4.3.1 Temperature and Rainfall

Mycotoxins exhibit distinct geographical variations globally, attributed to the varying optimal production temperatures and a_w_ values of *Aspergillus* fungi. In Northern Italy, the occurrence of drought conditions and increased temperatures has led to considerable contamination of maize grain with *A. flavus* and AFs, subsequently resulting in the introduction of AFM1 into the dairy supply chain.^[^ ^111^ ^]^ Zhang et al.^[^ ^112^ ^]^ has suggested that *A. flavus* underwent an extensive transcriptomic response to variations in available water, transitioning from freely available water to water stress. Furthermore, elevated temperature and extreme dryness experienced in 2012 led to the presence of aflatoxins in 69% of maize samples collected from Serbia.^[^ ^113^ ^]^ The temperature range that leads to OTA production varies among fungal species: *A. niger* and *A. carbonarius* have optimal growth temperatures of 30–35 °C and 25–30 °C, respectively.^[^ ^114^ ^]^ Furthermore, humidity can enhance OTA production even at reduced temperatures; at moderate temperatures ≈20 °C and with a water activity of 0.96–0.98 a_w_, the formation of OTA is notably increased.^[^ ^115^ ^]^ It is crucial to recognize that with rising temperatures, more hazardous mycotoxins are likely to proliferate, with AF, for instance, gaining ascendancy over OTA as the predominant mycotoxin. This is attributable to the fact that temperatures will be increasingly conducive to the proliferation of thermotolerant *Aspergilli*, which are the primary producers of AF.^[^ ^116^, ^117^ ^]^ Fumonisins production is believed to peak at approximately 30 °C.^[^ ^118^ ^]^ Although *Fusarium* species can tolerate higher temperatures, they are not as xerotolerant as *A. flavus*. Consequently, in regions where rainfall is expected to decline, fumonisins may be supplanted by aflatoxins.^[^ ^119^, ^120^ ^]^

The expression of mycotoxin biosynthetic genes in various mycotoxigenic fungi was found to be enhanced when subjected to the combined abiotic stresses of water activity and temperature.^[^ ^121^ ^]^ Gallo et al.^[^ ^122^ ^]^ discovered that *aflD* and *aflO* were elevated in tandem with increased production of AFB1 when they investigated the effects of the interaction between two factors (a_w_ x Temp.) on the growth of *A. flavus*. Further research has revealed that four genes within the aflatoxin biosynthetic pathway (*aflS, aflR, aflB*, and *aflT*) exhibited a 2.2–5.1 log2 fold increase in their expression levels when exposed to a a_w_ of 0.91 at 37 °C compared to 30 °C. This suggests that critical genes in the aflatoxin cluster are more responsive to water stress conditions (lower water availability) at elevated temperatures.^[^ ^123^ ^]^

Previous research has investigated the interactions between water stress and temperature on the relative expression of biosynthetic genes associated with the production of mycotoxins. Research evaluating the use of antioxidants under varying conditions, such as temperature, a_w_, and controlled atmospheres, have been conducted as potential methods to mitigate the growth of *Aspergillus* and the production of mycotoxins. However, the issue at hand is that the expense associated with these treatments is likely to persist in being too high for widespread adoption.

#### 4.3.2 CO_2_ Level

The escalating levels of atmospheric CO_2_ have significantly contributed to global warming, leading to numerous adverse effects that impact food production and increase mycotoxin contamination in crops, fruits, and vegetables.^[^ ^124^ ^]^ It has been reported that the increased of *A. alternata* spore production is associated with elevating CO_2_ levels.^[^ ^125^ ^]^ Research indicated that the production of AFB1 was notably elevated under the influence of climate change‐related factors, as opposed to current conditions (350–400 versus 1000 ppm CO_2_).^[^ ^123^ ^]^ Similarly, in an additional study, the impact of CO_2_ levels (400 versus 1000 ppm) on AFB1 production was assessed, revealing that stimulation occurred at the higher concentration of 1000 ppm.^[^ ^126^ ^]^ The previous study has confirmed that when the *laeA, veA*, and *velB* genes are upregulated, exposure to 1000 ppm CO_2_ can directly affect the global regulatory velvet complex, thereby influencing OTA production.^[^ ^127^, ^128^ ^]^ In addition, the epigenetic reader SntB has been demonstrated to regulate PAT biosynthesis, and may react to post‐harvest storage conditions, including low temperatures and high levels of CO_2_.^[^ ^117^ ^]^

The rapidly changing climate introduces significant variability, complicating the reliance on mycotoxin research data for any specific factor. The control of mycotoxin production remains a process, which is far from being fully understood to this day. In general, the relevant regulations are influenced not only by genomic regulators but also by climate change. The existing mathematical models are not yet comprehensive, additional physical and biological factors must be investigated.

## 5 Analytical Techniques for *Aspergillus* Mycotoxins Detection

Mycotoxin detection technology and quantitative methods have been continuously upgraded and reported in recent years. An assay method characterized by its rapidity, sensitivity, and specificity is essential for the routine analysis of foods, beverages, and feed. The common methods including chromatographic techniques, immunological techniques, and biosensor technologies, are listed below.

### 5.1 Chromatographic Techniques

Chromatographic methods enable the precise separation of analytes, thereby facilitating a comprehensive purification process. Subsequently, the purified analytes are subjected to quantification using a variety of detection techniques. Chromatographic methods employed in the analysis of mycotoxins encompass thin‐layer chromatography (TLC), high‐performance liquid chromatography (HPLC), gas chromatography‐mass spectrometry (GC‐MS), and liquid chromatography‐mass spectrometry (LC‐MS). By coupling to detectors, such as ultra violet (UV) detector, diode array (DAD), visible detector, fluorescence detectors (FLD), the compounds separated by chromatography could be further identified.^[^ ^129^ ^]^

#### 5.1.1 Thin‐Layer Chromatography (TLC)

TLC is a form of liquid chromatography characterized by a liquid mobile phase and a stationary phase that comprises a thin layer of material coated onto a flat substrate. It is also one of the most versatile methods of isolating the specific compounds in mixtures because of its affordability, ease of use, rapid development duration, elevated sensitivity, and strong reproducibility.^[^ ^130^ ^]^ TLC involves both adsorption and partitioning, as they are governed by similar types of intermolecular forces, including induced dipole‐dipole interactions and hydrogen bonding.^[^ ^131^ ^]^ For example, the OTA content is detected lower than 2 µg L^−1^ by using TLC method in wine.^[^ ^132^ ^]^ High performance thin layer chromatography (HP‐TLC) and reverse‐phase thin‐layer chromatography (RP‐TLC) are used to analyzed the mycotoxins content and confirm results. Although the TLC equipment is cheap and simple, there are still a few factors that affect its reproducibility and precision. Nowadays, TLC methods have been integrated with immunoaffinity techniques to improve detection.^[^ ^133^ ^]^

#### 5.1.2 High‐Performance Liquid Chromatography (HPLC)

HPLC is a widely utilized technique for the detection and quantification of various mycotoxins in contaminated materials. Its significance in mycotoxin analysis is attributed to its exceptional sensitivity, reproducibility, and accuracy.^[^ ^134^ ^]^ Its separation was worked by an RP‐18 column.^[^ ^135^ ^]^ However, the method is costly. Primarily the intricating sample preparation and the need for standards are complex.

#### 5.1.3 Liquid Chromatography‐Mass Spectrometry (LC‐MS)

The MS technique is being utilized with growing frequency for the detection of mycotoxins. The LC‐MS technique has emerged in recent years as the preferred method for the multi‐residue analysis of mycotoxins present in our environment.^[^ ^136^ ^]^ As a high‐precision detection method, LC‐MS has many advantages, including rapid analysis, high detection limits, reliability, and suitability for automation. Mycotoxin samples are introduced into the sampling system, from which they subsequently proceed to the chromatographic column for the purpose of separation. Following this, the samples are directed to the mass spectrometer for detection. Finally, the fragmented ion peaks would be analyses.^[^ ^137^ ^]^ However, it is worth noticing that the methods are usually high‐cost, time‐consuming and need trained testers. Besides, it could not provide more detailed structural information of the molecules.

#### 5.1.4 Gas Chromatography‐Mass Spectrometry (GC‐MS)

GC‐MS‐based metabolomics is an excellent method for identifying and quantifying small‐molecule metabolites (less than 650 Da), including mycotoxins.^[^ ^138^, ^139^ ^]^ GC‐MS detection has been reported to be widely useful as a confirmatory method for several mycotoxins due to its high chromatographic resolution, sensitivity, and accuracy. Zhang et al.^[^ ^140^ ^]^ established the GC‐MS PLS‐DA (partial least squares‐discriminant analysis) model, which could be used to classify the *A. carbonarius* strains that secreted the low content of OTA in the grapebased medium. The primary distinction of GC‐MS compared to other metabolomic analytical platforms (including LC‐MS), is the requirement for the derivatization.^[^ ^139^ ^]^ Otherwise, GC‐MS can analyze a wide range of compound classes using a single type of column. The electron ionization mode facilitates the fragmentation of molecules based on their structures, which aids in their identification.^[^ ^141^ ^]^

### 5.2 Immunological Techniques

Immunoassays that rely on immunological principles have been widely used for mycotoxins detection around the world, because of their sensitivity, high specificity, on‐site and high‐throughput screening ability.^[^ ^142^ ^]^ The outcome is contingent upon the interaction between an antigen, which serves as the target analyte, and a specific antibody that has been isolated and developed in response to that antigen. Antibodies have several isotypes such as IgG, IgM, IgE, IgD, and IgA that are with specific binding sites and different from each other in terms of the immune response. The main antibody‐based immunoassays are radioimmunoassay (RIA), enzyme‐linked immune sorbent assays (ELISAs), and lateral flow immunoassay (LFA).

#### 5.2.1 Radioimmunoassay (RIA)

The immunoassay methodology is founded on the principle of competitive binding between a radioactively labeled antigen and a nonradioactive counterpart. In this process, the radioactively labeled antigen competes with the nonradioactive antigen for a fixed quantity of immunoglobulins. This interaction involves an unknown concentration of unlabeled antigen and a predetermined amount of labeled antigen, which engage with a known and restricted concentration of immunoglobulin in standardized solutions. In peanuts, the AFB1 was detected by solid phase RIA in 1970s.

#### 5.2.2 Enzyme‐Linked Immune Sorbent Assays (ELISAs)

Based on antigen antibody‐specific binding for immune response, ELISAs offer the advantage of delivering rapid quantitative and semi‐quantitative analytical results, and they have been extensively utilized in the analysis of mycotoxins.^[^ ^143^ ^]^ They tested commercial kits for the important mycotoxins. Over the years, different types of ELISAs were developed to improve the specificity and sensitivity, and they could be divided to the direct assay, indirect, sandwich, competitive, and nanoparticle‐based ELISA. Nowadays, ELISA has been used for the simultaneous detection of five common mycotoxins including AFB1 and OTA in human serum. However, the matrix effects caused the extract limitation, which could arise from numerous factors such as other co‐extracted compounds, extract pH, extraction solvent composition, and sample processing. Based on these, the samples have to be pretreated before through the ELISA kits, examples include the purification of extracts, the dilution of extracts, or the incorporation of detergents.^[^ ^143^ ^]^ Consequently, the elevated costs and restricted storage stability of antibodies limit the applicability of these rapid analytical techniques.^[^ ^144^ ^]^

#### 5.2.3 Lateral Flow Immunoassay (LFA)

LFA, or named immunochromatographic strip test, is a point‐of‐care device. The underlying technical principle of LFAs is that a liquid sample containing the analytes or their extracts traverses various regions of a strip via capillary action.^[^ ^145^ ^]^ The fundamental LFA is a paper‐based system that comprises sample coating pads, conjugate‐release pads, absorbent pads, and membranes, which are also referred to as detection pads.^[^ ^146^ ^]^ LFAs can be further classified into sandwich and competitive types based on the different detection methodologies employed. In the sandwich format, the analyte binds to both the detector probe and the capture probe, making it particularly suitable for the detection of large molecules through the use of three distinct types of antibodies. While the competitive format is more suitable for small molecules by using just two kinds of antibodies.^[^ ^146^ ^]^ LFA is a more simplified immunoassay and the result could be read‐out within several minutes.^[^ ^147^ ^]^ The method doesn't need sophisticated equipment and skillful operators, and it could be applied outside the laboratory.^[^ ^148^ ^]^ Based on these, simple‐to‐use, low‐cost, and high‐speed help the LFA could be more widely used in the detection of the mycotoxins. However, LFAs have still been plagued by issues such as poor accuracy and semi‐quantification.^[^ ^147^ ^]^ In recent years, various integrated techniques, including fluorescence resonance energy transfer (FRET), aptamers, and surface‐enhanced Raman spectroscopy (SERS) in conjunction with LFAs, have been proposed and implemented. They have greatly contributed to the widespread development of LFA.

In light of the progress made in contemporary scientific research and the rigorous standards established for food safety and public health, there is a pressing need to lower the detection limits of various mycotoxins. The integration of immunological technology with test paper into portable devices represents a significant initiative that warrants implementation.

### 5.3 Biosensor Techniques

A biosensor can be broadly defined as a measurement device that incorporates a biologically derived sensing element that is closely integrated with a sensor component. Compared with the tomographic and immunological techniques discussed above, the favorable properties of the biosensor methods include portability, sensitivity, ongoing analytical capabilities, and a low cost associated with each analysis.^[^ ^149^ ^]^ Mycotoxins or antibodies are the most frequently used bio‐recognition element. Based on the variety of bio‐recognition elements, biosensors can be classified into immunosensors, aptasensors, and molecularity imprinted polymer (MIP)‐based sensors, enzymatic inhibition, and mimotope.^[^ ^150^, ^151^ ^]^ The sensors are also can be categorized in two groups like labeled and label‐free sensors. In addition, the sensor can be electrochemical (EC), optical, mass‐sensitive, and piezoelectric as well. Several types would be introduced in detail below.

#### 5.3.1 Labeled and Label‐Free Immunosensors

The labeled immunosensor can be categorized into two types: competitive and non‐competitive, based on the detection strategy employed.^[^ ^152^, ^153^ ^]^ Labeled non‐competitive immunoassays frequently employ a sandwich format, wherein the secondary antibody, which is tagged with enzymes, fluorescent materials, or nanoparticles, produces a signal upon the capture of the antigen.^[^ ^154^ ^]^ In this methodology, the sample analyte engages in competition with a coated labeled analyte or an analyte‐protein conjugate for a limited quantity of antibody binding sites.^[^ ^155^, ^156^ ^]^ A significant advantage of label‐free immunosensors lies in their simplicity and the ability to operate without the need for reagents in a single‐stage process.

#### 5.3.2 Aptasensors

The aptasensor is composed of an aptamer, which is a synthetic oligonucleotide ligand, either in the form of single‐stranded DNA or RNA, characterized by the presence of 10 to 50 variable nucleotide bases. They could exhibit high specificity and strong binding affinity.^[^ ^157^ ^]^ In comparison to conventional instrumental methods and immunological techniques, aptamer‐based biosensor technologies have been effectively developed for the detection of a range of mycotoxins, demonstrating high sensitivity and selectivity. Commonly used labels are fluorescence material, enzyme HRP, and electroactive compounds (such as ferrocene, ferro‐cyanide, methylene blue, and other metal nanoparticles). Recent advancements in the field of biosensing have led to the development of label‐free aptasensors for the detection of OTA utilizing the thickness shear mode acoustic method (TSM)^[^ ^158^ ^]^ and surface‐enhanced Raman spectroscopy (SERS).^[^ ^159^ ^]^ Additionally, Guo et al.^[^ ^160^ ^]^ have successfully engineered a real‐time quantitative polymerase chain reaction (RT‐qPCR) based aptasensor specifically for the detection of AFB1.

#### 5.3.3 Molecularity Imprinted Polymer (MIP)‐Based Sensors

MIPs are artificial materials designed to mimic biological receptors. They are produced via the polymerization of functional monomers in the presence of a specific target analyte. MIPs have garnered significant interest as recognition elements in sensor development due to their high selectivity and several advantageous properties, including ease of preparation, physical and chemical robustness during use, reusability, stability, and the potential for large‐scale production.^[^ ^161^ ^]^ The inaugural electrochemical sensor utilizing MIPs was developed in the 1990s.^[^ ^162^ ^]^ More recently, Pacheco et al.^[^ ^163^ ^]^ designed a straightforward electrochemical sensor for the detection of OTA by modifying a glassy carbon electrode (GCE) with MIPs. This combined sensor demonstrated a LOD of 4 × 10^−9^ mol L^−1^.

#### 5.3.4 Electrochemical (EC) Sensors

EC biosensors are sophisticated instruments characterized by their high sensitivity, which is achieved by amplifying a signal via the use of enzyme labels. Potentiometric and amperometric are the most popular methods among the group of electrochemical biosensors. Potentiometric biosensors operate by monitoring specific ions as indicators at the interface, resulting in a condition where the electric current at the electrode is zero (I = 0). In contrast, amperometric biosensors detect variations in current (I ≠ 0) that are attributed to the activity of the enzyme label. Electrochemical biosensors have been employed for different mycotoxins. For example, AFB1 was detected by electrochemical array immunosensor, and the LOD was 30 pg mL^−1^ in indirect competitive format.^[^ ^164^ ^]^

#### 5.3.5 Optical Sensors

Optical biosensors utilize antibodies that are either conjugated with fluorochromes or exhibit inherent fluorescence properties associated with certain mycotoxins. The main advantages of optical methods are sensitivity to optical contamination and stability, and can be used numerous times.^[^ ^165^ ^]^

#### 5.3.6 Mass‐Sensitive Sensors

Mass‐sensitive sensors, commonly known as electroacoustic sensors, utilize electroacoustic technology, which involves the conversion of acoustic energy into electrical energy, or the reverse process. The advancement of quartz crystal microbalances (QCM) for biosensing applications is fundamentally based on the piezoelectric effect demonstrated by quartz crystals.^[^ ^166^ ^]^ A QCM transducer is composed of a thin disk of quartz crystal situated between two gold electrodes, with one electrode being functionalized to detect the specific analyte of interest. QCM could provide real‐time analysis and high sensitivity for the detection of the mycotoxins. Besides, it's slao easy to operate and offers portability to small transducers.

Biosensors designed for the detection of mycotoxins should be regarded as a promising analytical approach. Development of a fitted biosensor for mycotoxins analysis in place of chromatography and ELISA, is a thrust area in the field of biosensor study. Future research may focus on the development of multi‐analysis detection systems utilizing lab‐on‐a‐chip platforms that are cost‐effective.

Different methods have different characteristics. Given that, the application of the real‐time data and sensing strategies, analytical techniques would grow more mature for detecting mycotoxin substances. Although numerous analytical techniques have been developed over last decades, mycotoxin analysis continues to represent huge challenges to researchers. The present methods focus more on preparing optimized antibodies with better specificity, strip materials enhancement, and structure design. The applications of leading‐edge techniques are really encouraged to extend from the agriculture field to the dinner table at an early date. For instance, the smartphone has become a commonly utilized device in contemporary society, whether the combination of smartphone and analytical techniques could offer enabling instrument miniaturization and complexity of detection, while with highly sensitive and quantitative detection, so that we could eat more healthy each meal. No doubt, more sensitive and specific detection methods research would greatly contribute to the safety control in agricultural products, food, and human health in the future.

## 6 Management Strategy for *Aspergillus* Mycotoxins Contamination

**Figure 6:**
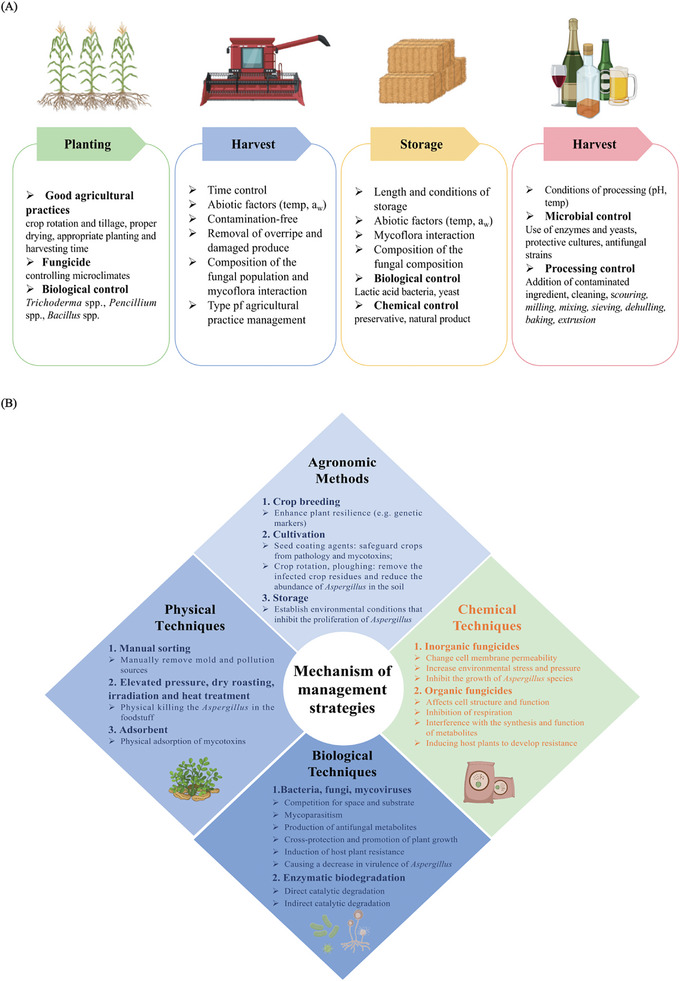
A) Factors and practices that may affect the concentrations of mycotoxins in cereals throughout the production and processing continuum. B) A concise diagrammatic representation summarizing the mechanisms of pertinent management strategies.

The contamination of food and feed by mycotoxins is a risk for human resulting in various destroyed regarding health status. A series of practices could be implemented to avoid the entry of mycotoxins and to minimize their frequency in food products from the planting to proceeding stages (**Figure** **6**A). Given that a single management strategy usually cannot fully control *Aspergillus* mycotoxin contamination, various strategies for elimination and preventing the risks of mycotoxins contamination are listed below.

### 6.1 Agronomic Methods

Agronomic prevention strategies against mycotoxin contamination consist of pre‐harvest and post‐harvest prevention methods. The particular mechanisms can be categorized into three distinct areas: enhancing plant resilience, safeguarding plants from harm, and eradicating mold and its associated pathogens from the environment (Figure 6B).

Prior to the harvest of crops, strategies for the prevention of mycotoxins are primarily implemented during the cultivation phase in the field. One effective approach to mitigate mycotoxin contamination in crops is through crop breeding. Over the past decades, genetic markers such as RFLPs, SSRs, and SNPs, have been implemented in maize enhancement initiatives, specifically for the purposes of marker‐assisted and genomics‐assisted breeding aimed at developing resistance to ear rot, which is induced by the *Aspergillus* species.^[^ ^167^ ^]^ During planting time, cultivation management also plays an important role in pre‐harvest mycotoxin control. Conventional seed coating agents frequently incorporate hazardous substances that pose a risk to environmental integrity and subsequently endanger human health. The new environment‐friendly seed coating agents have characteristics of disease prevention and keep the seeds off the mycotoxins.^[^ ^168^ ^]^ Crop rotation with proper species is effective in decreasing the mycotoxins, such as legumes and wheat.^[^ ^169^ ^]^ Ploughing, minimum tillage, no‐tillage technology, and other proper practice of soil cultivations could contribute to the removal of the infected crop residues, which lead to the reduction of mycotoxin risks invading the crops.^[^ ^169^ ^]^ It is worth noticing that improper supplies of water and nutrition might foster the soil stress, and further may cause the mycotoxin accumulation.

Storage represents a critical phase in the post‐harvest management of mycotoxins. Within this context, the interplay of various biotic factors, including grains, bacteria, yeasts, and fungi, alongside abiotic factors such as moisture, air, and temperature, is significant. Among these, temperature and humidity emerge as the most pivotal determinants influencing fungal infection and subsequent mycotoxin contamination. It is generally observed that maintaining a water activity level below 0.7 can effectively mitigate mold spoilage.^[^ ^170^ ^]^ Seed‐coating formulation has also developed these years. For instance, the incorporation of the 10–15% DCF RL‐1‐178 into the formulation for maize grain coating has the potential to mitigate mycotoxin levels during storage for a duration of at least five months.^[^ ^171^ ^]^ Mathematical models have rapid developing for regional mycotoxin prediction on field scale.^[^ ^172^ ^]^ Nevertheless, the relationships between the occurrence of mycotoxins, climatic variables, and other biological factors remain inadequately understood, and existing modeling systems exhibit limitations that require enhancement.^[^ ^173^ ^]^

### 6.2 Physical Techniques

Implementation of optimal agricultural methodologies, manufacturing techniques, appropriate storage conditions, and any other preventions are not always effective in decreasing mycotoxins contamination.^[^ ^174^ ^]^ Traditional cleaning method means to remove the large dust and pick out the inferior grains by human labor.^[^ ^175^ ^]^ Van der Westhuizen et al.^[^ ^176^ ^]^ reported that manual sorting has contributed to enhancing product safety by removing over 90% of various types of foreign unwanted materials (FUM). However, it is high dependent on the experiences of workers and only could not be carry out large‐scale activities for efficiency and high costs.

Steam subjected to elevated pressure, dry roasting, irradiation, and heat treatment all have been found to be effective in controlling mycotoxins contamination in crops.^[^ ^177^ ^]^ While the decomposition temperature of AFs can reach as high as 280 °C, the application of dry heating processes may mitigate the effects of mycotoxins. Specifically, *A. flavus* and its associated mycotoxins, AFG1 and AFB1, can be significantly reduced, with reductions of up to 83%, following one day of treatment with pulsed electric fields (PEF) over treatment durations ranging from 0.5 to 24 h.^[^ ^178^ ^]^ In treated grape juice, high hydrostatic pressure (HPP) and PEF treatments are significantly to reduce AF levels.^[^ ^179^ ^]^ AFB1 has relatively high sensitiveness to gamma irradiation. At a dose of 6 kGy of gamma irradiation, the AFB1 level would be reduced in peanuts.^[^ ^180^ ^]^ At a dose above 10 kGy of gamma irradiation, the AFB1 concentration might significantly decrease in soybeans.^[^ ^181^ ^]^ Besides, irradiation of contaminated samples also showed high efficiency in reducing OTA. Maatouk et al.^[^ ^182^ ^]^ reported that the concentration of OTA could significantly reduced by 62% in contaminated millet flour byirradiation at a dose of 4 kGy. Gamma irradiation at a dosage of 15 kGy, exposure to sunlight for a duration of 20 h, and UV irradiation for 12 h have been shown to nearly eliminate OTA in maize grain. Furthermore, UV irradiation at a wavelength of 222 nm demonstrates the greatest efficacy in reducing PAT levels in apple juice. Additionally, heat treatments at temperatures of 100 °C and 150 °C for 90 min led to substantial reductions in AFB1 concentrations in soybean, with decreases of 41.9% and 81.2%, respectively, and it confirmed that long‐time heat treatment has a beneficial on decontamination.^[^ ^185^ ^]^ PAT could be broken down to minor molecules, such as 3‐keto‐5‐hydroxypentanol, subsequent to prolonged exposure to elevated temperatures.^[^ ^186^ ^]^

Adsorbent is another technique used for *Aspergillus* and mycotoxins control. Synthetic polymers, zeolites, complex carbohydrates, and activated charcoal could be used normally. In addition, some gasses could also be used to reduce the mycotoxins. For example, Weng et al.^[^ ^187^ ^]^ showed that aqueous NH_4_OH could decrease AFB1 by 99.8%. After that, a study by Allameh et al.^[^ ^188^ ^]^ observed that negative consequences associated with aflatoxin‐contaminated maize may be mitigated through the application of ammonia treatment, specifically utilizing vapors containing 1% ammonia. Except that, Torlak et al.^[^ ^189^ ^]^ reported a method controlling AFB1 levels of 86.4% in feed, by ozone treatment for 6 h. The rate of degradation of PAT in juice is influenced by the duration of ozonation, with observed reductions in PAT concentration from 201.06 µg L^−1^ to below 50 µg L^−1^ occurring within a 15‐minute period.^[^ ^190^ ^]^ Meanwhile, the high cost of ozonation might limit their applications.

Based on these, some shortcomings that limit the physical methods applicating in food and feed manufacturing also should be discussed. Both extra costs of equipment and chemical safety of ionizing irradiation could not be ignored in productive practice.^[^ ^191^ ^]^ Besides, some proteins are sensitive to irradiation, which could lead to changes in taste of milk.^[^ ^192^ ^]^ These methods are not ideal for mycotoxins reduction in food and feed.

### 6.3 Chemical Techniques

Some chemicals are helpful to decrease mycotoxins for their high efficiency and low cost. In practice, the fungicides including inorganic and organic compounds, were often used to control fungal infections in crops, fruit and vegetables. Research indicates that acids, alkalis, oxidizing agents, and aldehydes can inhibit the growth of *Aspergillus* species. Specifically, sodium bisulfite, calcium hydroxide, formaldehyde, sodium hypochlorite, and sodium borate have demonstrated a significant capacity to lower aflatoxin levels in a variety of food products.^[^ ^193^ ^]^ For instance, a control rate of 68% for aflatoxin was achieved through the application of heat at 65 °C following a 1‐h treatment with sodium bisulfite.^[^ ^194^ ^]^ Furthermore, the use of various salts, acids, and alkaline compounds (consist of hydrochloric acid, phosphoric acid, sodium hydroxide, potassium hydroxide, calcium hydroxide, sodium bicarbonate, sodium chloride, and sodium sulfate), showed to control AFs contamination by as much as 18–51%.^[^ ^194^ ^]^ Antioxidants such as butylated hydroxyanisole, also demonstrated significant efficacy in the inhibition of *A. flavus*, resulting in a decrease in aflatoxin contamination. The effectiveness of fungicides in reducing OTA is contingent upon the specific type of fungicide used and the timing of its application. For instance, carbendazim (methyl‐2‐benzimidazol carbamate) is a commonly utilized fungicide that is instrumental in the management of OTA levels. But it is worth noticing that too much carbendazim would promote *A. carbonarius* predominance and might lead to OTA increased.^[^ ^195^ ^]^ In addition to this, the fungicides Switch and Mikal were also demonstrated to be effective in decreasing fungal colonization and the content of OTA.^[^ ^118^, ^196^ ^]^

Actually, the chemical methods for reducing mycotoxins also have their own disadvantages. The European Commission has prohibited the use of chemical treatments for the reduction of mycotoxins in feed and food products,^[^ ^197^ ^]^ and the related researches were gradually less published recently years.

### 6.4 Biological Control Techniques

Compared with the chemical fungicides, microorganisms have a relatively narrow spectrum of activity and are usually considered as plant protection products.^[^ ^198^ ^]^ Biological control technique is an environmental‐friendly strategy that uses living organisms or their derivatives to control mycotoxins. The biological control agents (BCAs) are mainly atoxigenic strains consist of beneficial bacteria, fungi and mycoviruses. The mechanisms of BCAs include competition for space and substrate, mycoparasitism, production of antifungal metabolites, cross‐protection, promotion of plant growth, induction of host plant resistance (Figure 6B).^[^ ^199^ ^]^ The application of atoxigenic strains of *Aspergillus* as a biocontrol measure against toxigenic strains has proven effective in mitigating the production of mycotoxins in agricultural settings.

Atoxigenic strains require a high ratio of atoxigenic strains compared to toxigenic strains. When the atoxigenic strains are applied in the field, they will compete with the toxigenic strains, and mitigating the proliferation of toxigenic fungi to decrease aflatoxin levels.^[^ ^200^ ^]^ Chiotta et al.^[^ ^201^ ^]^ evaluated a native non‐aflatoxigenic strain of *A. flavus*, designated AFCHG2, had been employed in field trials to mitigate aflatoxin production in peanuts. Additionally, the atoxigenic strain BN30 has demonstrated efficacy in controlling aflatoxin contamination in maize across various regions in Africa.^[^ ^202^ ^]^ In our country, *A. flavus* AF051 has showed high effectiveness in reducing aflatoxin in peanut fields, which was up to 99%.^[^ ^203^ ^]^ In Argentina, Ponsone et al.^[^ ^204^ ^]^ reported that the Kluyveromyces thermotolerans strains have demonstrated the ability to regulate the growth and accumulation of OTA produced by strains within the *Aspergillus* section *Nigri*. Given that the transcript level of *otapks* gene was not completely congruent with OTA accumulation, transcriptional regulation of the *otapks* might be responded to antagonistic *K. thermotolerans*.^[^ ^204^ ^]^

At the experimental stages, several yeast stains including *Debaryomyces hansenii* strain BCS003, *Kluyveromyces* spp., *Pichia anomala*, *Candida maltose*, *Saccharomyces cerevisiac* RC008, and *Saccharomyces cerevisiae* RC016, show a drastic effect on aflatoxins with the inhibition to the growth of *Aspergillus* spp.^[^ ^205^ ^]^ In addition, *Trichoderma* spp. and *Pencillium* spp. are considered important biocontrol agents among pathologies fungi. Research indicates that *Trichoderma* species have effectively diminished AFs contamination in groundnuts and sweet corn by as much as 57% and 65%, respectively.^[^ ^205^ ^]^ Beyond that, many bacterial species have also shown inhibition of mycotoxins by inhibition the growth of *Aspergillus* spp. *Bacillus* spp., *Pseudomonas* spp., *Lactobacillus* spp., and *Streptomyces* spp. are main species among them. Kong et al.^[^ ^206^ ^]^ even found that *Bacillus megaterium* completely inhibited the production of AFs in broth medium, achieving a 100% reduction. Generally, *B. megaterium*, *B. subtilis*, *B. amyloliquefaciens*, *B. mojavensis*, *B. cereus*, *B. mycoides*, and *B. pumilus* are considered as the most effective decreasing aflatoxin levels in *Bacillus* species.

Enzymatic biodegradation of mycotoxins is a crucial technology for their control. Carboxypeptidase A was initially extracted from the pancreas of cattle in 1969.^[^ ^207^ ^]^ The enzyme facilitates the hydrolysis of the amide bond linking the amino acid L‐phenylalanine (Phe) to the isocoumarin residue of OTA, resulting in the formation of Phe and OTα. The toxicity of OTα is significantly lower than that of OTA. Natural detoxification of OTA to Phe and OTα in the gastrointestinal tract of ruminants is highly efficient.^[^ ^208^ ^]^ Schmidt et al.^[^ ^209^ ^]^ isolated an aflatoxin‐degrading enzyme from *Armillariella tabescens* and expressed it in the embryo tissue of developing maize kernels. The transgenic maize lines demonstrated markedly lower concentrations of aflatoxin 30 days following infection, in comparison to non‐transgenic counterparts, even when subjected to *A. flavus* AF13 strain.

Biological control presents a viable strategy for mitigating the risks associated with mycotoxins. The application of biological control methods for managing plant pathogens serves as a complementary approach to chemical control within the context of integrated pest management (IPM), thereby contributing to the reduction of mycotoxin prevalence in food supply chains.^[^ ^198^ ^]^ However, the registration procedure of biocontrol agents is highly costly. Besides, compared with the spent of chemical pesticides, biocontrol agents are a relatively little investment in the research. More studies are needed in the future.

## 7 Conclusions and Future Perspectives

Aflatoxins and ochratoxins represent two primary categories of secondary metabolites produced by *Aspergillus*. Despite concerted efforts to minimize the levels of *Aspergillus* mycotoxins at every stage of food and feed product processing, the prevalence of these mycotoxins remains alarmingly high globally. Our paper reviewed the impact of *Aspeigillus* mycotoxins on food safety and human health. Despite our utmost efforts to prevent crop diseases, *Aspergillus* mycotoxins continue to pose a threat to our food security and the health of both animals and humans.

Advanced and diverse technologies have empowered humanity to focus more intently on the structures and biosynthetic pathways of mycotoxins, employing a range of methodologies. It could assist them in concentrating on the regulators of *Aspergillus* and mycotoxins, potentially offering valuable insights for developing countermeasures to prevent or mitigate harm to humans. Furthermore, we delved into the effects of climate change on the contamination of *Aspergillus* mycotoxins. Whereas now, rapid climate change brings more challenges, relying on mycotoxin research data to understand specific factors. It is worth considering and conducting in‐depth research on the transfer pathways of mycotoxins from fields to human foods and their ultimate impact on human health under current climate change scenarios.

Advanced and innovative detection technologies, along with novel management strategies, have been implemented globally to combat *Aspergillus* and associated mycotoxins. To be candid, these strategies also possess certain drawbacks, including high costs and low efficacy, which necessitate overcoming. Indeed, the potential economic benefits of *Aspergillus* mycotoxin contamination on industrial workers must be taken into account in practical terms. Complete elimination of *Aspergillus* mycotoxins from the environment is not feasible, but we are committed to minimizing their presence to prevent adverse effects on human health. We propose that biocontrol methods should be integrated with other physical and chemical strategies, with a particular emphasis on reducing *Aspergillus* contamination and the accumulation of mycotoxins. To produce safe and healthy agricultural products, it is essential to consider economic, social, and environmental sustainability comprehensively. To ensure advancements in the field, the following points about *Aspergillus* mycotoxins are critical:

1. The concept of “Source Control” should be ingrained. Cultivating crops resistant to *Aspergillus* and mycotoxins, and implementing a more comprehensive variety management strategy throughout the field, storage, and transportation processes, would significantly mitigate the risk of mycotoxin ingestion by both animals and humans.
2. Encouragement and support should be given to foundational research exploring the interaction between crop growth and *Aspergillus* mycotoxins. Various molecular pathways, such as health effects of multiple mycotoxins, and the related functional genes are not clear. More resources must be devoted to foundational research that can clarify the responding mechanism of *Aspergillus* spp. and mycotoxins under changing environment.
3. Place greater emphasis on the influence of climate change on *Aspergillus* and mycotoxin contamination. Current scientific evidence and simulation models indicate that as climate change intensifies, plant disease pressures will markedly escalate, thereby adversely affecting food safety. Considering the complexity of climate change, emerging tools such as genomics, satellite imagery, digital technologies, big data, and machine learning should be integrated and leveraged to facilitate the control of *Aspergillus* and mycotoxins. We call for the establishment of the global surveillance network integrating artificial intelligence for data synthesis from field facilities and remote sensing. They might further enable early warnings of mycotoxins threats to humans.

## Conflict of Interest

The authors declare no conflict of interest.

## Supporting information



Supporting Information
