# Supplementary material for: Aspergillus Mycotoxins: The Major Food Contaminants
Source: Adv Sci (Weinh). 2025 Feb 7;12(9):2412757. doi: 10.1002/advs.202412757 (PMC11884562; doi:10.1002/advs.202412757)

**Table S1** Chemical structures of aflatoxins and the varieties of aflatoxin production in *Aspergillus* species.

| Types | Chemical Structures (a-e) | | | | | Producers |
| --- | --- | --- | --- | --- | --- | --- |
|  | Structure | R1 | R2 | R3 | R4 |  |
| AFB1 | a | H | OCH_3_ | =O | H | *A. flavus, A. arachidicola, A. bombycis, A. miniscierotigenes, A. nomius, A. ochraceoroseus, A. paraiticus, A. pseudotamarill, A. rambellii, A. pseudocaelatus, A. pseudonomius, A. texensis* |
| AFB2 | ac | H | OCH_3_ | =O | H | *A. flavus, A. arachidicola, A. miniscierotigenes, A. nomius, A. paraiticus, A. pseudocaelatus, A. texensis* |
| AFB2a | ad | H | OCH_3_ | =O | H | *A. flavus, A. niger* |
| AFB3 | f | - | - | - | - | *A. flavus* |
| AFG1 | b | H | - | - | - | *A. flavus, A. arachidicola, A. miniscierotigenes, A. nomius, A. paraiticus, A. pseudocaelatus, A. texensis* |
| AFG2 | bc | H | - | - | - | *A. flavus, A. arachidicola, A. miniscierotigenes, A. nomius, A. paraiticus, A. pseudocaelatus, A. texensis* |
| AFG2a | bd | H | - | - | - | *A. flavus, A. paraiticus* |
| G1 |  |  |  |  |  | *A. flavus, A. paraiticus* |
| AFGM2 | bd | OH | - | - | - | *A. flavus, A. paraiticus* |
| AFGM2a | bd | OH | - | - | - | *A. flavus* |
| AFM1 | a | OH | OCH_3_ | =O | H | *A. flavus, A. paraiticus* |
| AFM2 | ac | OH | OCH_3_ | H | H | *A. paraiticus* |
| AFM2a | ac | OH | OCH_3_ | H | H | *A. flavus* |

**Table S2** Chemical structures of OTA and its derived metabolites and varieties of the production in *Aspergillus* species.

| Types | Chemical Structures (h-i) | | | | | | Producers |
| --- | --- | --- | --- | --- | --- | --- | --- |
|  | Structure | R5 | R6 | R7 | R8 | R9 |  |
| OTA | h | Phenylalanine | Cl | H | H | H | *A.affinis, A. albertensis, A. alliaceus, A.carbonarius, A. cretensis, A. flocculosus, A. lacicoffeatus, A. niger, A.ochraceus, A. pseudoelegans, A. roseoglobulosus, A. sclerotioniger, A. sclerotiorum, A. steynii, A. sulphureus, A. westerdijkiae, Neopetromyces muricatus, P. nordicum,*  *P. verrucosum* |
| OTB | i | Phenylalanine | H | H | H | H |  |
| OTC | i | Ethyl-ester, phenylalanine | Cl | H | H | H |  |
| OTA Methyl-ester | i | Methyl-ester, phenylalanine | Cl | H | H | H |  |
| OTB Methyl-ester | i | Methyl-ester, phenylalanine | H | H | H | H |  |
| OTB Ethyl-ester | i | Ethyl-ester, phenylalanine | H | H | H | H |  |
| OTα | i | OH | Cl | H | H | H |  |
| OTβ | i | OH | H | H | H | H |  |
| 4-R-Hydroxyochratoxin A | i | Phenylalanine | Cl | H | OH | H |  |
| 4-S-Hydroxyochratoxin A | i | Phenylalanine | Cl | OH | H | H |  |
| 10-Hydroxyochratoxin A | i | Phenylalanine | Cl | H | H | OH |  |
| Tyrosine analog of OTA | i | Tyrosine | Cl | H | H | H |  |
| Serine analog of OTA | i | Serine | Cl | H | H | H |  |
| Hydroxyproline analog of OTA | i | Hydroxyproline | Cl | H | H | H |  |
| Lysine analog of OTA | i | Lysine | Cl | H | H | H |  |

**Table S3** Chemical structures of fumonisins and varieties of the production in *Aspergillus* and *Fusarium* species.

| Types | Chemical Structures (j-m) | | | | | | Producers |
| --- | --- | --- | --- | --- | --- | --- | --- |
|  | Structure | R10 | R11 | R12 | R13 | R14 |  |
| FA1 | j | OH | OH | H | NHCOCH_3_ | CH_3_ | *F. proliferatum*, *F. verticillioide, F. nygamai* |
| FA2 | j | H | OH | H | NHCOCH_3_ | CH_3_ | *F. proliferatum*, *F. verticillioide, F. nygamai* |
| FA3 | j | OH | H | H | NHCOCH_3_ | CH_3_ | *F. proliferatum*, *F. verticillioide, F. nygamai* |
| FAK1 | l | OH | OH | - | NHCOCH_3_ | - | *F. proliferatum*, *F. verticillioide, F. nygamai* |
| FBK1 | l | OH | OH | - | NH_2_ | - | *F. proliferatum*, *F. verticillioide, F. nygamai* |
| FB1 | j | OH | OH | H | NH_2_ | CH_3_ | *F. proliferatum*, *F. verticillioide, F. oxysporum, F. sacchari, F. fujikuroi, F. subglutinans, F. thapsinum, F. anthophilum, F. globosum, F. nygamai, F. dlamini, F. napiforme, F. pseudonygamai, F. andiyazi, F. oxysporum* var. *Redolens, F. polyphialidicum* |
| *Iso*-FB1 | j | OH | H | OH | NH_2_ | CH_3_ | *F. verticillioide* |
| FB2 | j | H | OH | H | NH_2_ | CH_3_ | *A. niger* NRRL 3122*, F. proliferatum, F. verticillioides* |
| FB3 | j | OH | H | H | NH_2_ | CH_3_ | *F. proliferatum, F. verticillioides* |
| FB4 | j | H | H | H | NH_2_ | CH_3_ | *A. niger* |
| FB6 | j | H | OH | OH | NH_2_ | CH_3_ | *A. niger* |
| NCM-FB1 | j | OH | OH | H | NHCH_2_COOH | CH_3_ |  |
| NDF-FB1 | j | OH | OH | H | NHDF | CH_3_ |  |
| HFB1 | m | OH | OH | - | - | - |  |
| HFB2 | m | H | OH | - | - | - |  |
| FC1 | j | OH | OH | H | NH_2_ | H | *F. proliferatum*, *F. verticillioide, F. nygamai, F. oxysporum* |
| *Iso*-FC1 | j | OH | H | OH | NH_2_ | H | *F. oxysporum* |
| FC3 | j | OH | H | H | NH_2_ | H | *F. oxysporum* |
| FC4 | j | H | H | H | NH_2_ | H | *F. verticillioide, F. oxysporum* |
| FP1 | k | OH | OH | - | - | - | *F. proliferatum* |
| FP2 | k | H | OH | - | - | - | *F. proliferatum* |
| FP3 | k | OH | H |  |  |  | *F. proliferatum* |

**Figure S1**. Structures, biosynthetic gene clusters, and proposed biosynthetic pathways of mycotoxins in *Aspergillus* species. (A) Chemical structures of aflatoxins (a-g), ochratoxins (h-i), fumonisins (j-m) and patulin (n). (B) The mycotoxin biosynthetic genes and gene clusters in Aspergillus. Arrows indicate position and transcriptional orientation of genes. Gene name is indicated near each arrow. (C) The proposed pathway of Aflatoxins B1 (AFB1) biosynthesis. (D) The proposed pathway of Ochratoxin A (OTA) biosynthesis. (E) The proposed pathway of fumonisins (FUM) biosynthesis. (F) The proposed pathway of patulin biosynthesis.


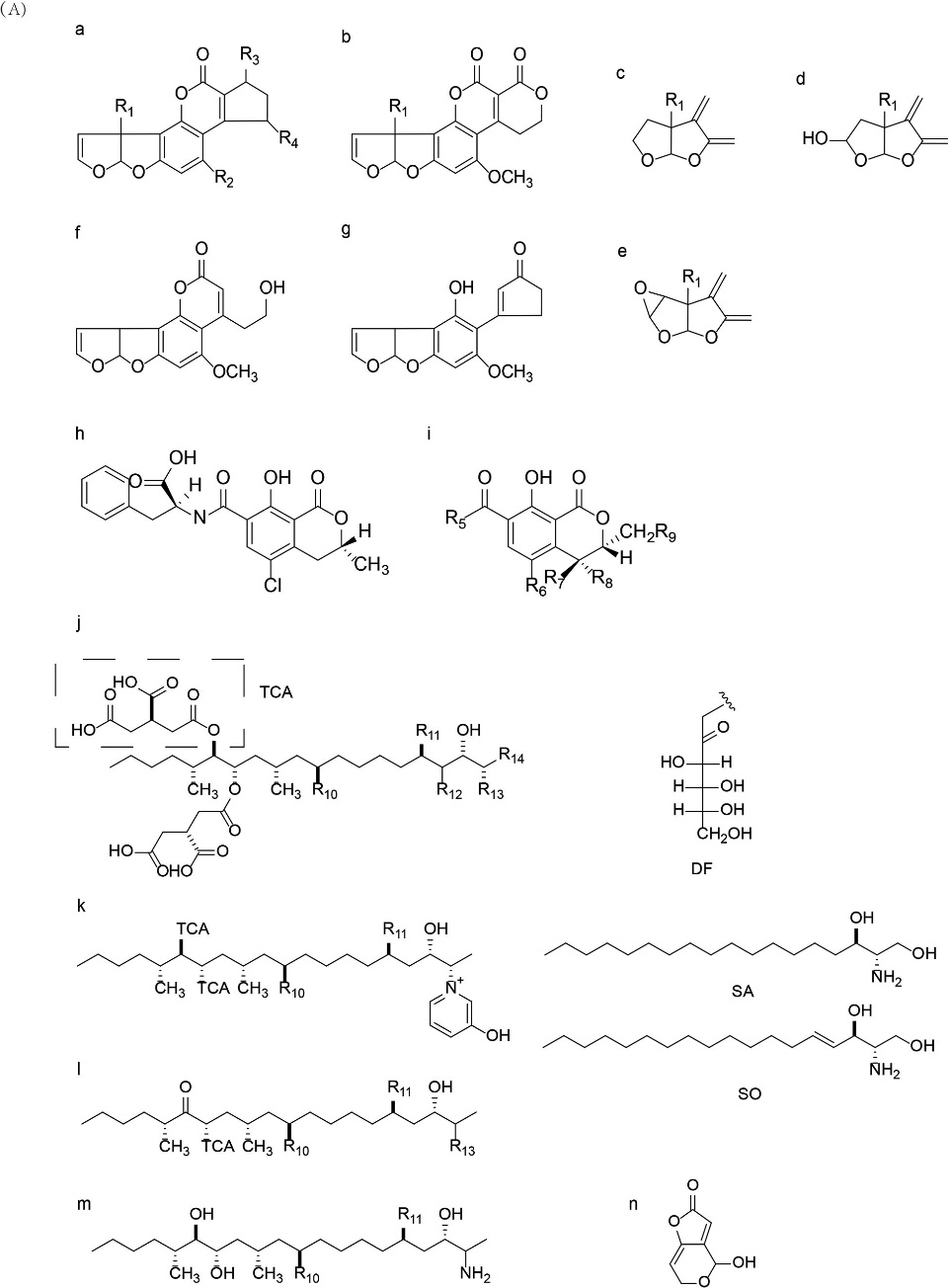


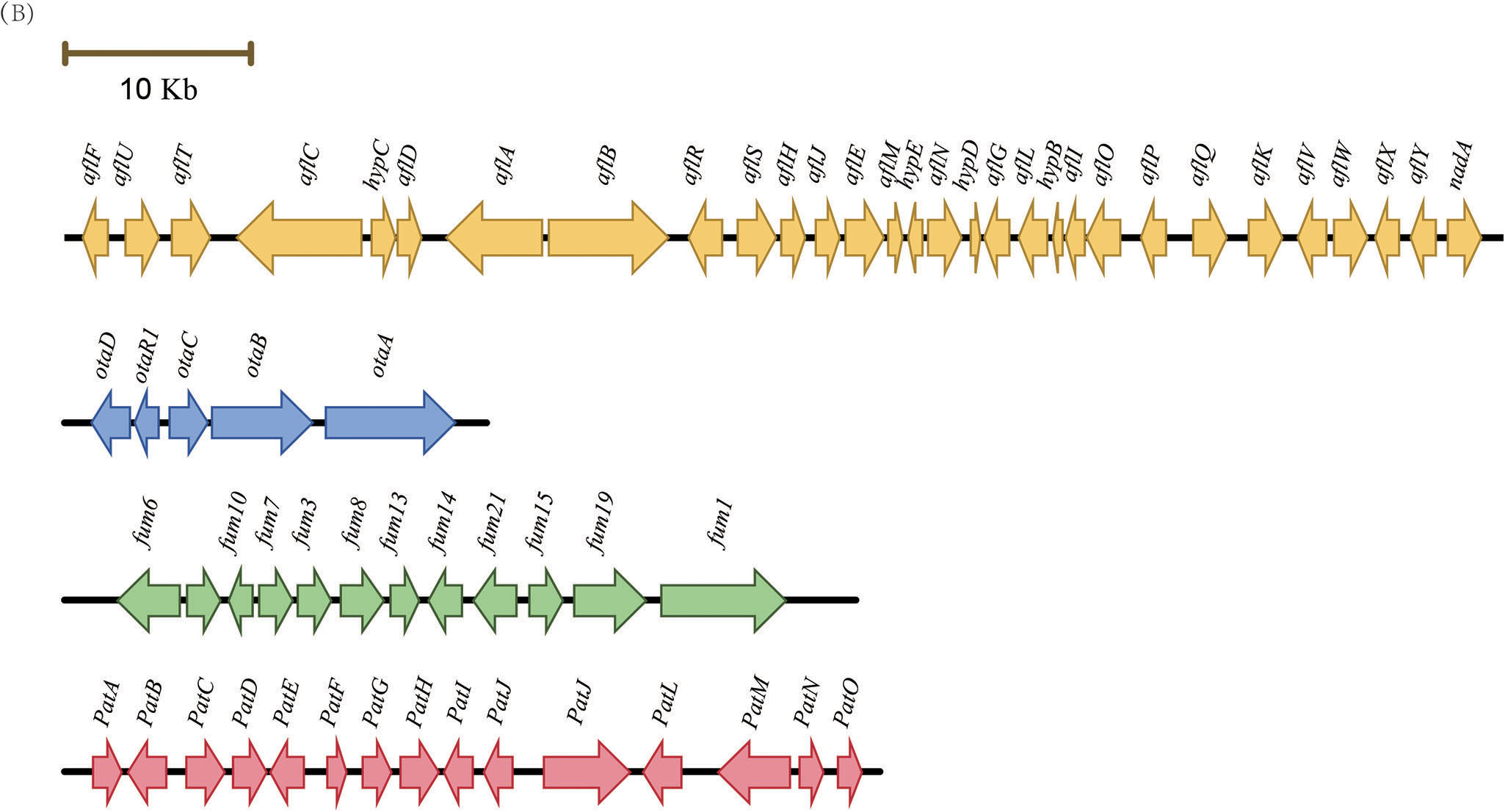


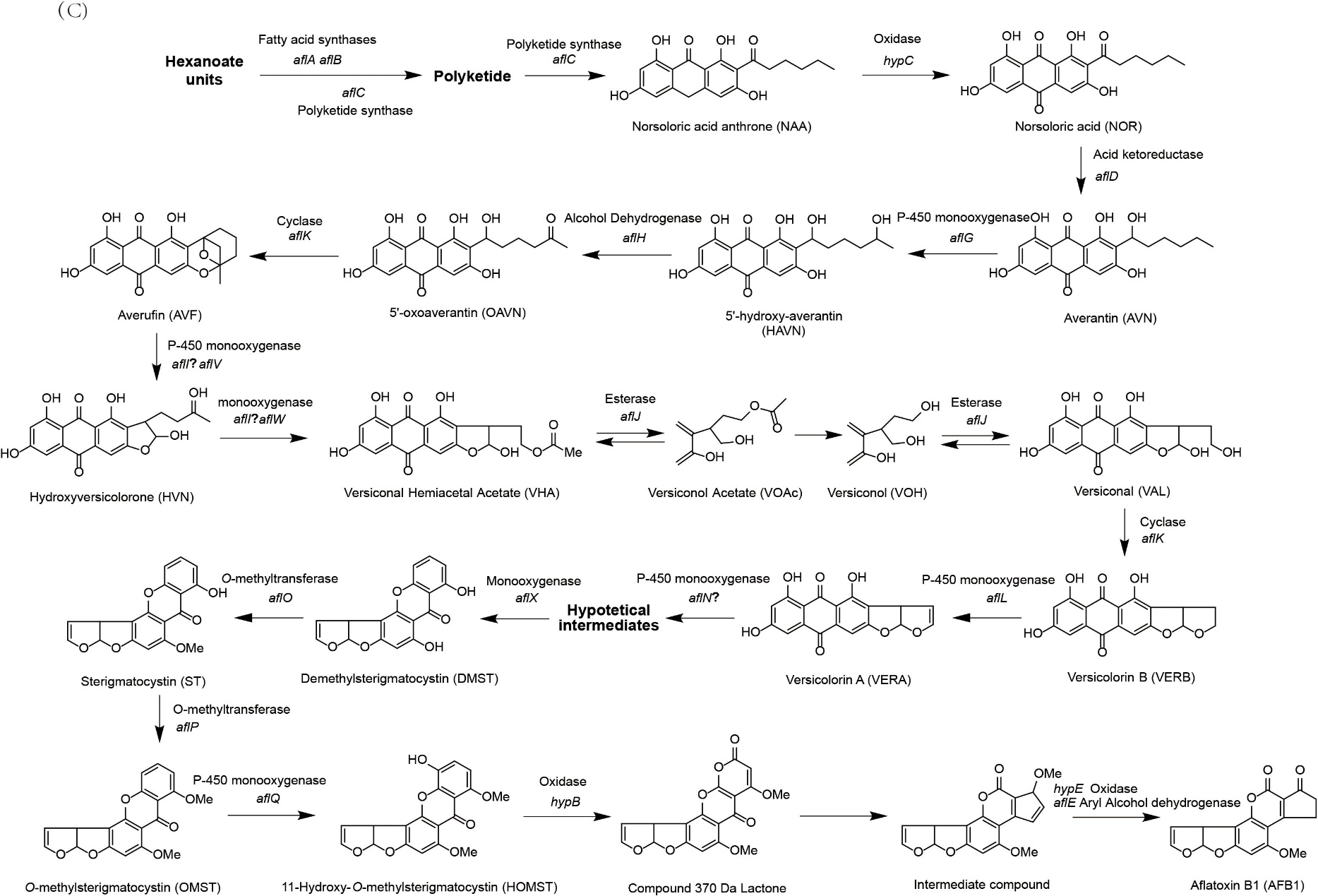


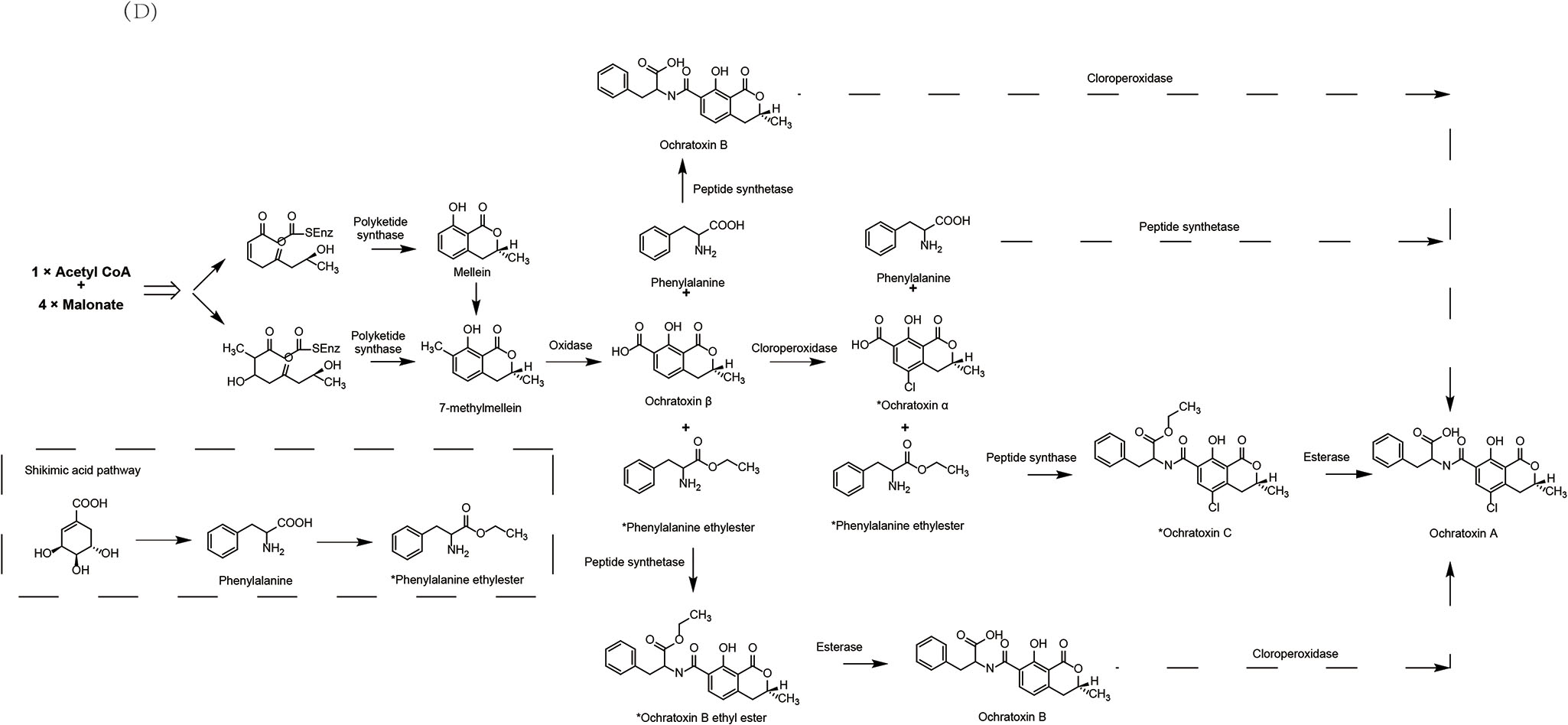


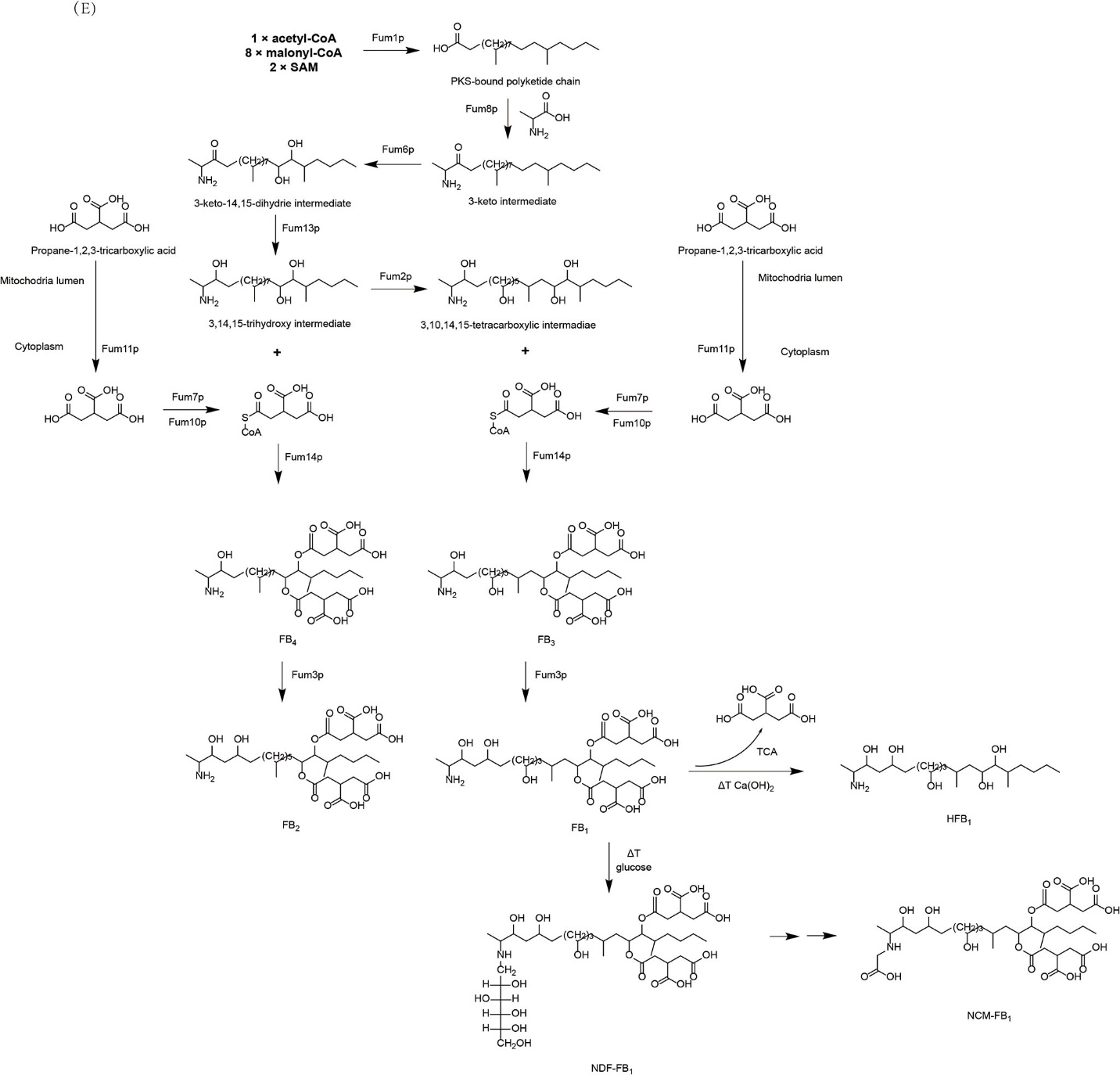


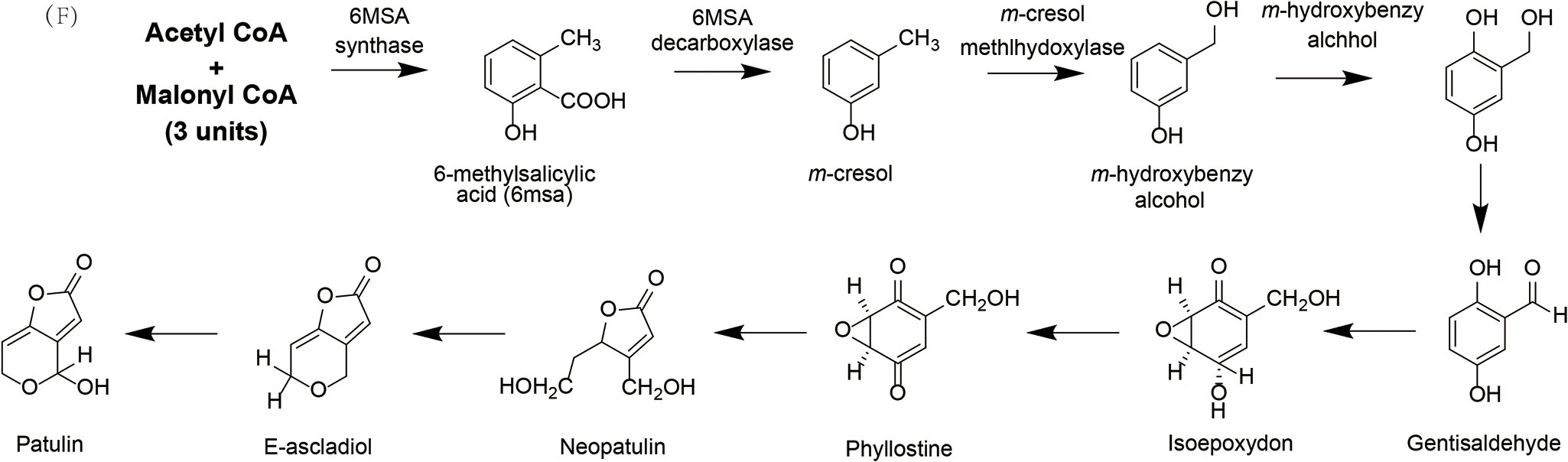

Supplement: Supplementary file 1 — Supporting Information [file ADVS-12-2412757-s001.docx]
